# Drug Administration Routes Impact the Metabolism of a Synthetic Cannabinoid in the Zebrafish Larvae Model

**DOI:** 10.3390/molecules25194474

**Published:** 2020-09-29

**Authors:** Yu Mi Park, Markus R. Meyer, Rolf Müller, Jennifer Herrmann

**Affiliations:** 1 Department of Microbial Natural Products, Helmholtz Institute for Pharmaceutical Research Saarland (HIPS), Helmholtz Centre for Infection Research (HZI) and Department of Pharmacy, Saarland University, Campus E8 1, 66123 Saarbrücken, Germany; Yu-Mi.Park@helmholtz-hips.de; 2 Environmental Safety Group, Korea Institute of Science and Technology (KIST) Europe, 66123 Saarbrücken, Germany; 3 Department of Experimental and Clinical Toxicology, Institute of Experimental and Clinical Pharmacology and Toxicology, Center for Molecular Signaling (PZMS), Saarland University, 66421 Homburg, Germany; m.r.meyer@mx.uni-saarland.de; 4 German Center for Infection Research (DZIF), Partner Site Hannover-Braunschweig Germany, 38124 Braunschweig, Germany

**Keywords:** zebrafish larvae model, metabolism, administration route, microinjection, HepaRG cells, mass spectrometry imaging (MSI), synthetic cannabinoid, methyl 2-(1-(5-fluoropentyl)-1*H*-pyrrolo [2,3-b]pyridine-3-carboxamido)-3,3-dimethylbutanoate (7′*N*-5F-ADB), 3R principle, drug metabolism and pharmacokinetics (DMPK)

## Abstract

Zebrafish (*Danio rerio*) larvae have gained attention as a valid model to study in vivo drug metabolism and to predict human metabolism. The microinjection of compounds, oligonucleotides, or pathogens into zebrafish embryos at an early developmental stage is a well-established technique. Here, we investigated the metabolism of zebrafish larvae after microinjection of methyl 2-(1-(5-fluoropentyl)-1*H*-pyrrolo[2,3-b]pyridine-3-carboxamido)-3,3-dimethylbutanoate (7′*N*-5F-ADB) as a representative of recently introduced synthetic cannabinoids. Results were compared to human urine data and data from the in vitro HepaRG model and the metabolic pathway of 7′*N*-5F-ADB were reconstructed. Out of 27 metabolites detected in human urine samples, 19 and 15 metabolites were present in zebrafish larvae and HepaRG cells, respectively. The route of administration to zebrafish larvae had a major impact and we found a high number of metabolites when 7′*N*-5F-ADB was microinjected into the caudal vein, heart ventricle, or hindbrain. We further studied the spatial distribution of the parent compound and its metabolites by mass spectrometry imaging (MSI) of treated zebrafish larvae to demonstrate the discrepancy in metabolite profiles among larvae exposed through different administration routes. In conclusion, zebrafish larvae represent a superb model for studying drug metabolism, and when combined with MSI, the optimal administration route can be determined based on in vivo drug distribution.

## 1. Introduction

Zebrafish (*Danio rerio*; ZF) has become a very prominent in vivo model organism in various research fields, such as toxicology, drug discovery, disease models, and neurobiology [1,2,3,4,5]. This has several reasons, such as ease of handling, predictivity of ZF assays, and their link to effects observed in humans. Importantly, the ZF genome shares 70% similarity to human genes and the similarity of potential human disease-related genes is even higher (82%) [6,7,8].

The use of self-feeding ZF embryos and larvae that are younger than 120 h post-fertilization (hpf) is particularly popular because such experiments are not considered as animal experiments according to European legislation (EU directive 2010/63/EU). Thus, experiments with larvae at ≤120 hpf are in compliance with the 3R principle (Replacement, Reduction, Refinement) as they contribute to the reduction of animal experiments. Consequently, ZF embryos and larvae have been widely applied in studies of e.g., human disease [9,10], infection [11,12], antibiotics [13,14], and human metabolism [15,16,17], to name just a few.

Microinjection has been used already for decades as a way of administering compounds to fish in early life stages, and this technique is still widely used in cellular microbiological research [18,19,20]. However, most laboratories use aquatic exposure in screening campaigns with ZF larvae, thus neglecting the potentially hindered uptake and absorption of lipophilic compounds which, in turn, can lead to a rather high rate of false negatives. More recently, some groups proposed to administer lipophilic compounds via microinjection into yolk sac due to easy and straightforward microinjection protocols that can be automated if required [21,22,23].

In an earlier study [24], we detected only one metabolite in ZF larvae after microinjection of a new psychoactive substance (NPS) into the yolk sac. In contrast, 18 metabolites were detected when we administered the compound to the ZF larvae through conventional aquatic exposure. This unexpected result prompted us to refine our protocols for metabolite identification in ZF larvae as part of our preclinical DMPK (drug metabolism and pharmacokinetics) assay pipeline.

The common sites for ZF microinjection are yolk sac, caudal vein, heart ventricle, and hindbrain. The corresponding techniques require different levels of expertise, with yolk sac injections being the simplest technique. However, more advanced microinjection techniques, such as caudal vein injections, provide the advantage of ensuring proper systemic distribution of a drug or other microinjected agents. For studying drug metabolism in ZF larvae, this appears to be crucial as test compounds need to reach the liver compartment where most metabolic reactions take place. Metabolic processing is mainly performed by cytochrome P450 (CYP) enzymes, and ZF larvae possess a full complement of CYP genes, which display functional similarity to the human orthologs. This similarity makes ZF a very promising model as its predictive value in comparison to human metabolism is thought to be high [25,26].

The spatial distribution of a drug can be checked by mass spectrometry imaging (MSI) as an outstanding visualization tool that enables label-free imaging in biological tissues. Here, we applied MSI in order to understand the distribution of a drug and its metabolites in ZF larval bodies, which, in turn, enables an informed choice on the optimal administration route. However, sample preparation of ZF larvae sections is still challenging.

**Figure 1:**
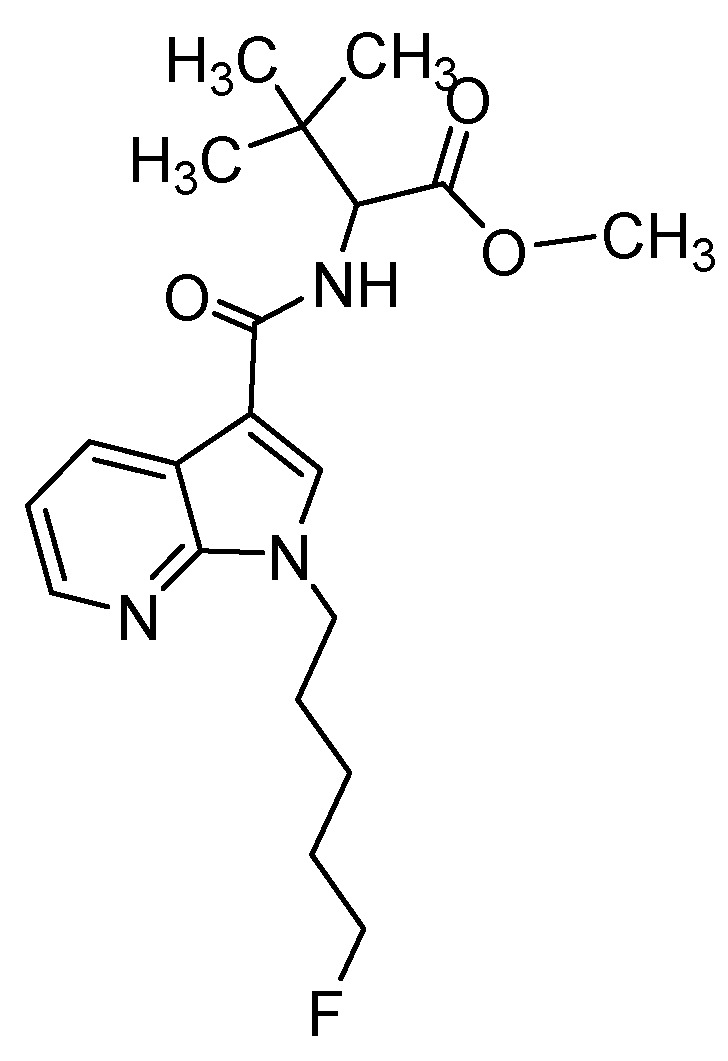
Chemical structure of 7′*N*-5F-ADB.

In this study, we present a new approach of employing various administration routes for drug exposure to ZF larvae to further improve metabolite identification resulting in a spectrum, which is widely in concordance with human metabolism. A new synthetic cannabinoid (SC), methyl 2-(1-(5-fluoropentyl)-1*H*-pyrrolo[2,3-b]pyridine-3-carboxamido)-3,3-dimethylbutanoate (7′*N*-5F-ADB, Figure 1), was chosen for this study because it appeared recently on the illicit drug market [27] as one of the most dangerous SCs with high potency and serious adverse effects resulting in hospitalizations and fatalities [28,29,30,31]. To characterize the circulation of 7′*N*-5F-ADB and its metabolites in the ZF larval body, the spatial distribution was visualized by matrix-assisted laser desorption/ionization (MALDI)-MSI as an emerging technique enabling the imaging of molecular species [32,33,34].

## 2. Results and Discussion

### 2.1. Zebrafish Larvae Metabolite Spectra of 7′N-5F-ADB Differ Depending on the Route of Administration

In a previous study [24], we investigated metabolites of 7′*N*-5F-ADB after addition of the compound to ZF larvae either through the larvae-surrounding water or through microinjection into yolk sac resulting in a reduction from 18 to only one detected metabolite when the yolk sac administration route was chosen. It is also worth mentioning that ZF larvae produced an authentic spectrum of metabolites as found in human biosamples after intake of 7′*N*-5F-ADB [27]. We initially expected that the lipophilic NPS would only be insufficiently taken up by the larvae when added to the water and that administration by microinjection should result in a larger number of detectable metabolites. This prompted us to investigate other injection sites and to compare ZF metabolism as part of the current study. Table 1 summarizes phase I and phase II metabolite data from microinjected larvae and HepaRG cells, the latter being used for comparison, along with data from previously published studies with 7′*N*-5F-ADB [24,27].

**Figure 2:**
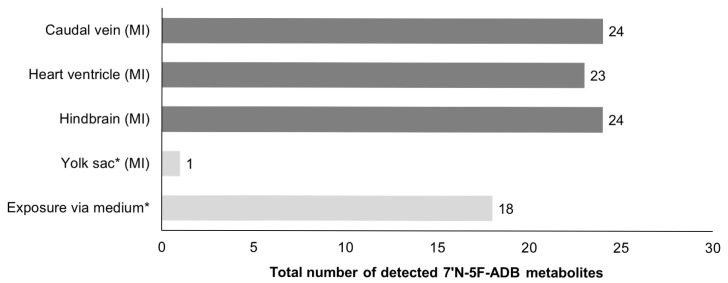
Comparison of the total number of metabolites produced depending on different administration routes in ZF larvae. Results for the samples investigated in this study are represented by the mean value of peak numbers of triplicates of 36 pooled larvae. (MI: microinjection, * these results were quoted from the previous study of 7′*N*-5F-ADB [24]).

**Figure 3:**
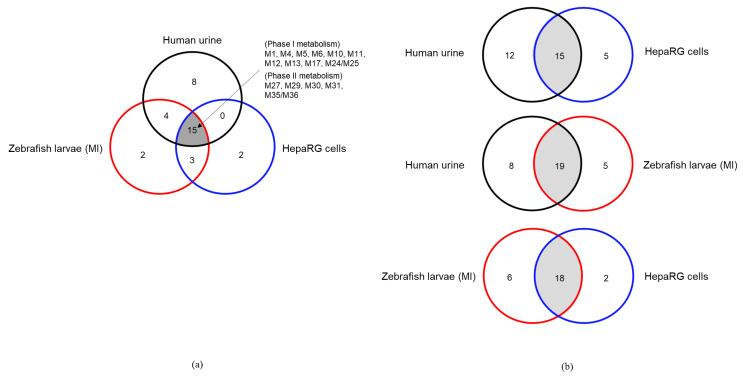
Comparison of the metabolites observed from the three models [human urine samples, zebrafish larvae (exposed via microinjection; MI), and HepaRG cells] using Venn diagrams. (**a**) Common metabolites detected among all models and (**b**) their mutual comparability.

Among the different models used (human, cells, zebrafish), a total number of up to 27, 20, and 24 metabolites was found in human urine, HepaRG cells, and microinjected (caudal vein, heart ventricle, hindbrain) ZF larvae, respectively (Table 1 and Supplementary Table S1). In contrast, the microinjection of 7′*N*-5F-ADB into the yolk sac of ZF larvae or addition of the compound to the water resulted in fewer detected metabolites (Figure 2). Importantly, out of the 24 metabolites from ZF larvae, 19 metabolites represent human metabolites from urine samples, three were also detected in HepaRG cells, and only two were exclusively found in the larvae (Figure 3). Reviewing the comparability of the three models, metabolite data of ZF larvae treated through microinjection showed high similarity to human urine samples, with a 70% match rate, and to HepaRG cells with a 90% match rate.

**Figure 4:**
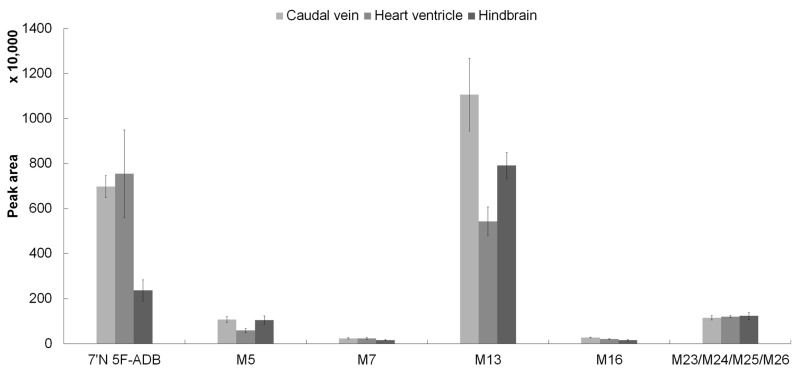
Detection profile of 7′*N*-5F-ADB and five major metabolites in microinjected ZF larvae (caudal vein, heart ventricle, and hindbrain). The most abundant metabolite, M13, was formed by defluorination in combination with oxidation of the formed primary alcohol (M7) to a carboxylic acid moiety. The clustered columns are displayed as mean ± SD (*n* = 3). The four structural isomers (M23, M24, M25, and M26) at *m/z* 410.2075 are represented as one metabolite due to co-elution from the LC-HRMS/MS system.

The most abundant metabolites of 7′*N*-5F-ADB in human urine samples [27] were M5 (ester hydrolysis), M11 (ester hydrolysis in combination with hydroxylation of the tertiary butyl part), and M30 (ester hydrolysis in combination with glucuronidation). In the ZF larvae model, M5, M13 (oxidative defluorination in combination with oxidation to carboxylic acid), and M23/M24/M25/M26 (four isomers; dihydroxylation of 7′*N*-5F-ADB) were the main metabolites (Table 2), and M13 was the fifth most abundant in human urine as well. Moreover, to study potential differences in metabolite patterns after microinjection of 7′*N*-5F-ADB into different organs of ZF larvae, we compared the five most abundant metabolites (Figure 4) and 17 minor metabolites (Supplementary Figure S1) detected in microinjected ZF larvae. Regarding the major metabolite M13, caudal vein injections were most successful as the respective peaks were detected at significantly higher abundance than in the other samples (heart ventricle and hindbrain injections). For the other major and minor metabolites, we did not observe specific patterns depending on the route of administration, but overall, metabolite peak detection was most feasible from samples where the compound was injected into the caudal vein. Yolk sac data could not be considered for the comparison due to only one metabolite (M13) being detected.

ZF larvae exposed to 7′*N*-5F-ADB via microinjection into different organs also produced three metabolites [M3 (*N*-dealkylation), M7 (defluorination), M15 (defluorination in combination with oxidation to carboxylic acid)] that were not detected in human biosamples (Table 1). In addition, M9 (ester hydrolysis with combination with defluorination and hydroxylation of the pentyl chain) was found only in all microinjected ZF larvae except yolk sac compartment, and its detection was reported in rat urine of a published study [27]. M2 was uniquely observed in only ZF larvae treated by exposure medium, and seven out of 10 phase II glucuronidated metabolites detected in human urine were produced in ZF larvae microinjected into three internal organs excluding yolk sac.

**Figure 5:**
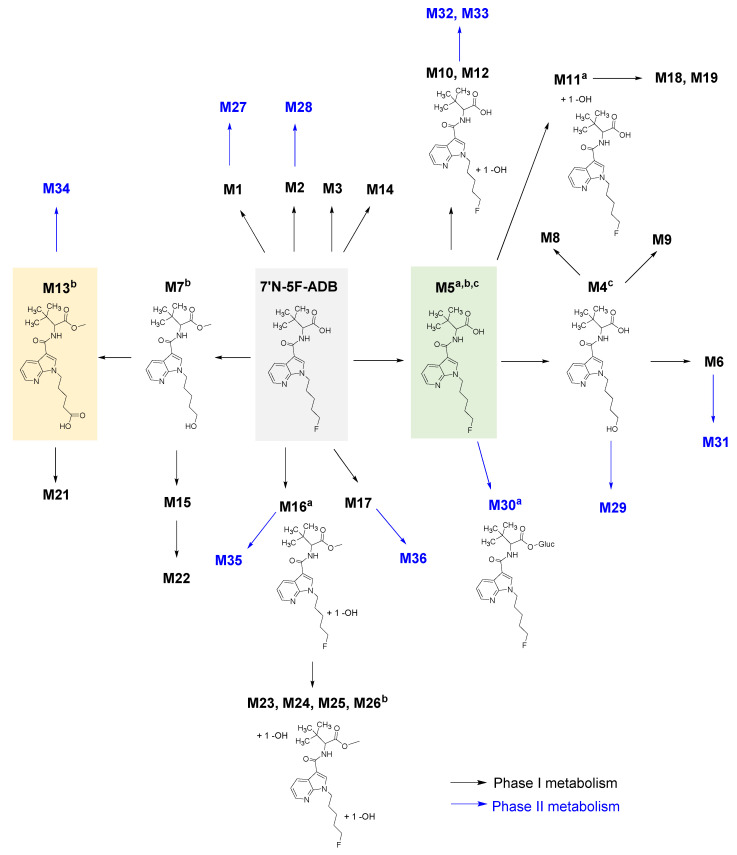
Overview of the reconstructed metabolic pathway of 7′*N*-5F-ADB based on the results from all investigated models. The progress of phase I metabolism is represented and the two major metabolites detected in all ZF larvae exposed to 7′*N*-5F-ADB, which were M5 and M13, are highlighted by a green and yellow box, respectively. Resulting phase II metabolites are displayed in blue. ^a^ Major metabolites in human samples; ^b^ major metabolites in microinjected ZF larvae; ^c^ major metabolites in HepaRG cells. A schematic representation of the reconstructed pathway from all three models can be found in Supplementary Figure S5.

The metabolic pathway of 7′*N*-5F-ADB was reconstructed with complementing the metabolites detected in all ZF larvae applied to different administration routes, including human urine samples [27] and HepaRG cells. The main pathways are displayed for phase I metabolism and phase II metabolism, which are centered on two abundant metabolites (M5, M13) found in the ZF larvae model (Table 2). Overall, we were able to construct the principal parts of the human metabolic pathway, as shown in Figure 5 based on the ZF larvae model.

Taken together, microinjection of 7′*N*-5F-ADB into caudal vein, heart ventricle, or hindbrain of ZF larvae resulted in a large number (24) of phase I and phase II metabolites that already formed as early as 1 h after compound administration with relatively high abundance. In contrast, detection of 18 metabolites after aquatic exposure with 7′*N*-5F-ADB required a much longer incubation period of 24 h. Microinjection into the yolk sac was not suitable for studying 7′*N*-5F-ADB metabolism in ZF larvae as demonstrated in a previous study [24]. Thus, direct injection into an internal organ of ZF larvae results in fast metabolism probably due to the fact that the NPS was able to reach functional metabolic organs, such as a liver and pancreas, through fast circulation inside of the larval body [35].

### 2.2. The Spatial Distribution of 7′N-5F-ADB in ZF Larva Is Visualized by MALDI-MSI

Mass spectrometry imaging (MSI) combined with MALDI enables coupling of high mass resolution data with visualized images of sample sections. Due to the results from metabolite detection following different administration routes for 7′*N*-5F-ADB into ZF larvae, we concluded that the drug distribution differs significantly if the NPS is given through the water, microinjected into yolk sac, or microinjected into caudal vein, heart ventricle, and hindbrain. Thus, MALDI-MSI was used to study the distribution of the parent compound 7′*N*-5F-ADB in the ZF larval bodies. To our knowledge, this is the first concise study of drug distribution in ZF larvae following different routes of compound microinjection.

**Figure 6:**
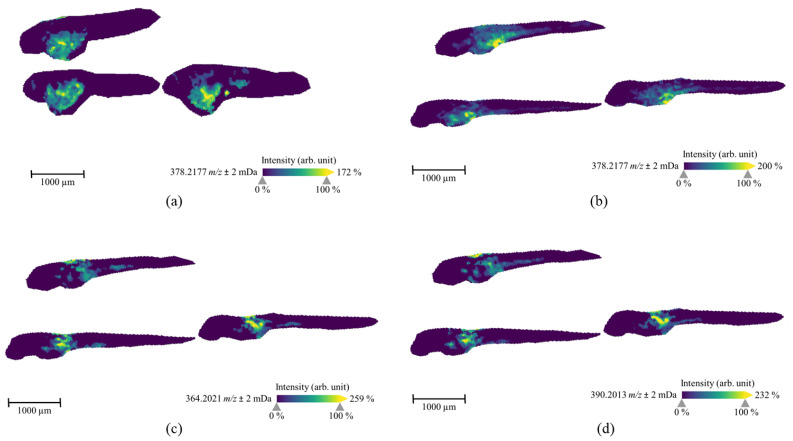
MALDI-MS images of the parent compound (7′*N*-5F-ADB, *m/z* 378.2177) in ZF larvae that were treated by aquatic exposure with 50 µM 7′*N*-5F-ADB for 1 d from 4 dpf to 5 dpf (**a**) or for 3 d from 2 dpf to 5 dpf (**b**). The MS images of two metabolites [M5, *m*/*z* 364.2021 (**c**) and M13, *m*/*z* 390.2013 (**d**)] visualized in the slices of B specimen. These metabolites were detected as the most abundant masses in all ZF larvae exposed via various administration routes. The presented sections originate from one representative larva per condition. The images were generated by preparing a colormap from blue (no detection) to yellow (high local concentration), and images were further processed by weak denoising in 96 dpi resolution with 24-bit color.

After 1-day exposure through medium, only 7′*N*-5F-ADB could be detected via MSI of sections of ZF larvae, and the parent compound exclusively accumulated inside the yolk sac. Therefore, we prepared ZF larvae that were treated for an extended period, from two days post-fertilization (dpf) to five dpf. In sections from these ZF larvae, we found the parent compound to be better distributed than in sections of one-day treated ZF larvae (Figure 6a,b). In summary, 7′*N*-5F-ADB and two major metabolites (M5 and M13; Figure 6c,d) appeared in dorsal and ventral regions at high abundances after 3 d treatment through aquatic exposure, but corresponding masses could not be detected in the tail end area of ZF larva.

**Figure 7:**
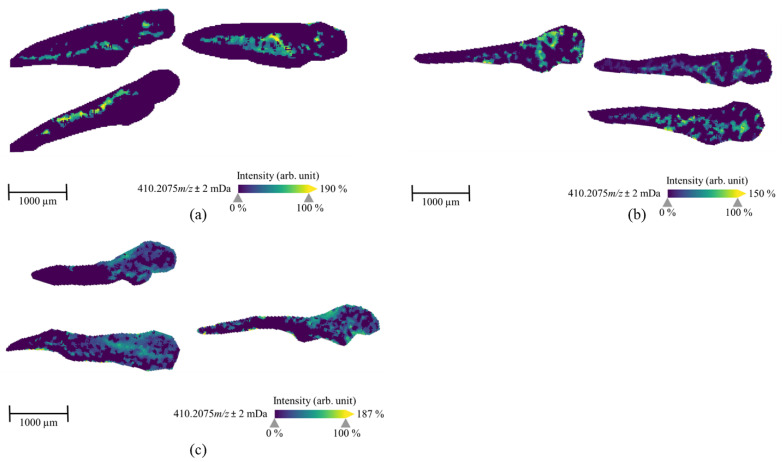
MALDI-MS images of the metabolite M23/M24/M25/M26 (four isomers, *m/z* 410.2075) in ZF larvae, which were exposed to 7′*N*-5F-ADB via microinjection into caudal vein (**a**), heart ventricle (**b**), and hindbrain (**c**) and then incubated at 28 °C for 1 h. These isomeric metabolites were the third most abundant in microinjected ZF larvae except ZF larvae treated through the yolk sac compartment. However, they cannot be differentiated in MSI due to lack of chromatographic separation. The presented sections originate from one representative larva per condition. The images were generated by preparing a colormap from blue (no detection) to yellow (high local concentration), and images were further processed by weak denoising in 96 dpi resolution with 24-bit color.

In contrast, it was not possible to generate distribution images of the parent compound and M13 in ZF larvae that were treated by microinjecting into caudal vein, heart ventricle, and hindbrain (Table 3), and M5 was identified in only one section among heart ventricle samples. This might be indicative of a fast distribution and an accordingly fast metabolism. We thus analyzed the spatial distribution of other major metabolites and compared their distribution to MSI data from ZF larvae that were exposed to 7′*N*-5F-ADB through water. We were able to image five metabolites from microinjected ZF larvae, all of which were structural isomers as determined by LC-HRMS/MS: M8/M9 (two isomers at *m*/*z* 378.2013), M10/M11/M12 (three isomers at *m*/*z* 380.1970), M16/M17 (two isomers at *m*/*z* 394.2126), M18/M19/M20 (three isomers at *m*/*z* 396.1919), and M23/M24/M25/M26 (four isomers at *m*/*z* 410.2075). The main metabolites that we could visualize by MSI are also listed in Table 3, and exemplary images of the isomers M23/M24/M25/M26 from larvae that were exposed through microinjection into different organs are depicted in Figure 7. Interestingly, depending on the route of administration, the images show distinct distribution patterns inside the larval bodies. In the caudal vein slices, the metabolite was detected along the dorsal aorta and concentrated in the veins and arteries of ZF larva. ZF larva microinjected into heart ventricle showed full spread of M23/M24/M25/M26 from head region to tail region, and high concentrations were found throughout the larval body. In contrast, hindbrain slices indicated the best distribution of this metabolite at intermediate concentrations. Images of these isomeric metabolites visualized by MALDI-MSI are represented as the summed distribution of the multiple isomers, as these constitutional and isobaric isomers cannot be distinguished due to lack of chromatographic separation in MSI. However, the presence of several isomers probably improved mass detection and imaging above the method detection limit (MDL).

In the MSI study, we observed a certain discrepancy when we compared the detection of metabolites from LC-HRMS/MS and MSI. Although MSI is a highly sensitive method at trace level, it could not detect the masses in some cases that were clearly detected by LC-HRMS/MS in pooled larvae samples. This effect was caused by the spatial distribution of lower abundant metabolites in the ZF larva and their further dilution due to the preparation of on average more than ten cryosectioned slices per larva. In particular, several metabolites, including M5 and M13, from microinjected ZF larvae were confirmed by LC-HRMS/MS, but it was not possible to generate MS images displaying a specific pattern. Further studies to improve the MDL of MALDI-MSI for the analysis of ZF larvae are ongoing. In summary, the treatment of ZF larvae with 7′*N*-5F-ADB through aquatic exposure resulted in a strong accumulation of the lipophilic NPS in the yolk sac, thus, protecting the compound from being metabolized in metabolically active organs. In contrast, when 7′*N*-5F-ADB was microinjected into vital organs of the ZF larvae, this resulted in the fast distribution and metabolism of the drug, as demonstrated by LC-HRMS/MS and MSI measurements. Furthermore, the finding was confirmed by the spatial distribution analysis of 7′*N*-5F-ADB and its metabolites in ZF larvae treated via microinjection.

We could demonstrate the importance of choosing the right administration route when studying drug metabolism in ZF larvae, also taking the chemical properties of the investigated compound into consideration. Here, the lipophilic nature of 7′*N*-5F-ADB hindered its proper distribution inside the larval bodies when given through water or when microinjected into the yolk sac. Intriguingly, we could observe a large number of (human) phase I and phase II metabolites at relatively high abundance when we microinjected the NPS into organs that support faster distribution, such as the heart ventricle. Moreover, the MS images showed distinct distribution patterns of its metabolites throughout the ZF larva body, which might be linked in future studies to potential toxic effects of compounds and their in vivo metabolites. Further studies are in progress to refine protocols for the cryosectioning of ZF larva and subsequent MSI experiments as it appears to be crucial to initially investigate compound distribution before proceeding to metabolite identification and to general pharmacological studies.

### 2.3. Comparison of Metabolite Identification in the In Vitro HepaRG Model and from ZF Larvae

The hepatic stem cell line HepaRG expresses cytochrome P450 biotransformation enzymes, phase II enzymes, and several transporters, which make it a prominent screening tool in toxicology and drug discovery studies. Due to the easy handling of the HepaRG cell line, it is a good alternative to primary human hepatocytes which are also often used as predictive *in vitro* model [36,37].

The metabolism of 7′*N*-5F-ADB in HepaRG cells was investigated referring to a previous study [24], and compared to the zebrafish larvae model. Furthermore, the time-dependent metabolism of 7′*N*-5F-ADB and formation of its metabolites was studied by analyzing cells after 0.1, 15, 60, 120, 240, 360, and 1,440 min of incubation. These time points were chosen based on waterborne exposure duration of ZF larvae (24 h) in order to study time-resolved metabolite formation within this period. HepaRG cells treated with 50 µM of 7′*N*-5F-ADB produced a total of twenty metabolites (Table 1). M5 formed by ester hydrolysis was the most abundant metabolite, which is in accordance to results from microinjected ZF larvae. M4 (ester hydrolysis in combination with oxidative defluorination), M10 and M11 (two isomers formed by ester hydrolysis in combination with hydroxylation of the tertiary butyl), and M30 (ester hydrolysis in combination with glucuronidation) were detected as the second-most abundant metabolites. However, M13, which was detected as a major metabolite in ZF larvae samples, was only found in minor amounts in HepaRG cells.

**Figure 8:**
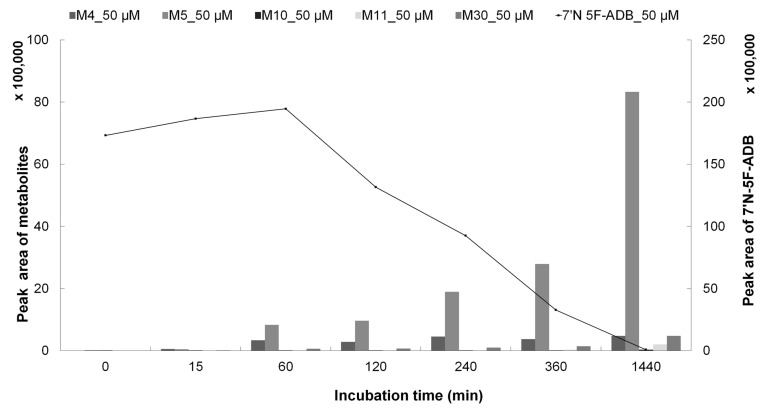
Internal amount-time profile of five main metabolites (M4, M5, M10, M11, and M30) in HepaRG cells incubated with 50 µM 7′*N*-5F-ADB (*n* = 2).

The concentration of 7′*N*-5F-ADB steadily increased up to 60 min and then declined significantly (Figure 8 and Supplementary Figure S2). Its metabolites had started to form markedly at this time point. Amounts of M5, M10, M11, and M30 were dramatically increased between 360 min and 1440 min, whereas M4 was increasing only slowly (Supplementary Figure S3).

Twenty metabolites could be produced in HepaRG cells with M5 and M4 being the most abundant metabolites. Interestingly, M22 (oxidative defluorination in combination with dihydroxylation of the pentyl chain) was found only in HepaRG cells, which was not detected in the human biosamples and ZF larvae samples. However, the detection of M22 was reported in rat urine samples of a published study [27]. In contrast, M23/M24/M25/M26, M28 (ester hydrolysis in combination with *N*-dealkylation and glucuronidation), and M34 (oxidative defluorination in combination with oxidation to carboxylic acid and glucuronidation) were not detected in HepaRG cells. The main metabolic pathway in HepaRG cells was constructed (Supplementary Figure S3) and 18 metabolites observed in HepaRG cells were also found in microinjected ZF larvae, corresponding to a 75% match rate. Fifteen metabolites from HepaRG were also detected in human urine samples, which corresponds to a 55% match rate (Table 1 and Figure 3). In conclusion, HepaRG cells constitute a valid *in vitro* model that can complement the in vivo ZF larvae model in drug metabolism studies.

## 3. Materials and Methods

### 3.1. Chemicals and Other Materials

7′*N*-5F-ADB was obtained from www.buyresearchchemicals.de tagged as 4′*N*-5F-ADB. However, NMR (nuclear magnetic resonance) studies confirmed it to be 7′*N*-5F-ADB. Dimethyl sulfoxide (DMSO), methylene blue, phenol red, tricaine (3-amino-benzoic acid ethyl ester), mineral oil, trifluoroacetic acid, gelatin from cold water fish skin, and 2,5-dihydroxybenzoic acid were obtained from Sigma-Aldrich (Taufkirchen, Germany). Methanol (LC-MS grade), acetonitrile (LC-MS grade), formic acid (LC-MS grade) were from VWR (Darmstadt, Germany). NaCl, KCl, MgSO_4_, Ca(NO_3_)_2_, and HEPES were obtained from Carl Roth (Karlsruhe, Germany). The 10 mM stock solution of 7′*N*-5F-ADB was prepared in DMSO and it was stored for a maximum of one month at −20 °C. The working solutions of 7′*N*-5F-ADB were freshly prepared prior to each experiment. Cell culture flasks, 24-well plates, and 6-well plates were purchased from Sarstedt (Nümbrecht, Germany). Basal hepatic cell medium MIL 700C and differentiation medium supplement with antibiotics ADD 720C were from Biopredic International (Saint-Grégoire, France). Glass capillaries TW100F-4 [4 inch (100 mm), 1/0.75 OD/ID (mm), Filament] were obtained from World Precision Instruments Germany GmbH (Friedberg, Germany). Undifferentiated HepaRG cells (HPR101) were purchased from Biopredic International (under MTA agreement No: 10528AHR10, Saint-Grégoire, France). Conductive indium-tin-oxide (ITO) coated glass slides were obtained from Bruker Daltonics (Bremen, Germany). ZF embryos of the AB wild-type line were initially obtained from the Luxembourg Center for Systems Biomedicine (Belvaux, Luxembourg). Dry small granulate food was purchased from SDS Deutschland (Limburgerhof, Germany), and *Artemia* cysts (>230,000 nauplii per gram) were obtained from Coralsands (Wiesbaden, Germany).

### 3.2. Zebrafish Maintenance and Embryo Collection

ZF husbandry and all experiments with ZF larvae were carried out in accordance with EU Directive 2010/63/EU and the German Animal Welfare Act (§11 Abs. 1 TierSchG). All works were performed following internal standard-operating procedures (SOPs) based on published standard methods [38].

Adult zebrafish for breeding were kept in an automated aquatic eco-system (PENTAIR, Apopka, UK). The following parameters are continuously monitored: Temperature (27 ± 0.5 °C), pH (7.0 ± 0.1), conductivity (800 ± 50 µs), and light-dark cycle (14 h/10 h). Fish were fed twice a day with dry small granulate food and additionally once a day with freshly hatched live *Artemia* cysts. The ZF embryo medium (0.3× Danieau’s solution) consisted of 17 mM NaCl, 2 mM KCl, 0.12 mM MgSO_4_, 1.8 mM Ca(NO_3_)_2_, 1.5 mM HEPES, pH 7.1–7.3, and 1.2 µM methylene blue. For ZF embryo production, AB wild-type line pairs were kept overnight in standard mating cages, separated by gender. In the following morning, the separators were removed, and the zebrafish spawned immediately. Fertilized eggs of zebrafish were selected using a LEICA M205 FA stereo microscope (Leica Mikrosysteme Vertrieb GmbH, Wetzlar, Germany), and embryos were raised in an incubator at 28 °C with daily medium change and cleaning of embryo cultures. ZF larvae at 4 dpf were used for drug metabolism studies.

### 3.3. Drug Treatment of ZF Larvae via Aquatic Exposure

The sample preparation following aquatic drug exposure is described in detail in a study by Richter et al. [24]. A non-toxic exposure concentration was chosen based on the survival rate as determined in in vivo maximum-tolerated concentration (MTC) experiments with 4 dpf ZF larvae. For metabolite studies, ten ZF larvae at 4 dpf were transferred to one well of a 6-well plate containing 3 mL of Danieau’s medium with 50 µM 7′*N*-5F-ADB and a final concentration of 1% (*v*/*v*) DMSO, and ZF larvae were treated for 24 h in an incubator at 28 °C. Additional ten larvae were incubated in compound-free medium containing 1% (*v*/*v*) DMSO that served as negative control (background masses). Prior to sample extraction, ten larvae were pooled, and sample extractions were performed as described below (Section 3.5). All samples were prepared in six replicates.

### 3.4. Drug Treatment of ZF Larvae via Microinjection into Different Compartments

The glass microneedle for microinjection was prepared by a Flaming/Brown type micropipette puller (Model P-100, Sutter Instrument, Novato, CA, USA) using the following settings: heat: ramp value ± 10, pull: 80, velocity: 60, delay time: 90 ms, and pressure: 13.8 bar. For injections, a 5 mM solution of 7′*N*-5F-ADB was prepared in 50% (*v*/*v*) DMSO and 50% (*v*/*v*) of a 0.5% phenol red solution. The injection needle was filled with 10 µL of 5 mM 7′*N*-5F-ADB without air bubbles by a microloader pipette tip and it was placed in a M-152 manipulator (Narishige Group, Tokyo, Japan) connected to a FemtoJet 4× Microinjector (Eppendorf, Hamburg, Germany). Before microinjection, all microinjection needles were calibrated by single droplet injections onto mineral oil on a micrometer slide. The injection volume (nL) was calculated according to the sphere volume equation (V = πγ^3^ 4/3) based on the diameter (mm) of the droplet [39,40]. We have chosen to microinject 4.19 nL of 5 mM 7′*N*-5F-ADB per larvae, which corresponds to a total amount of 284.4 ng in a pool of 36 larvae.

**Figure 9:**
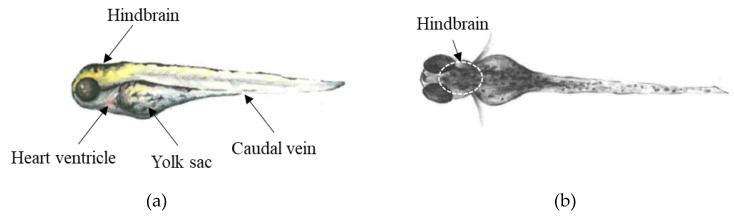
Microinjection sites used to administer 7′*N*-5F-ADB to 4 dpf ZF larvae in (**a**) lateral and (**b**) dorsal view.

ZF larvae at 4 dpf were anaesthetized by tricaine and then they were lined up on an agarose plate prepared using Z-MOLD (World Precision Instruments, Sarasota, USA). Excess medium was removed with a pipette. Microinjections were done into three different compartments of ZF larvae (caudal vein, heart ventricle, and hindbrain; Figure 9) under a stereo microscope (LEICA M205 FA stereo microscope). Larvae were directly transferred to fresh Danieau’s medium and they were incubated at 28 °C for 1 h. Prior to sample extraction, 36 larvae were pooled. Sample extractions were performed as described below (Section 3.5). All samples were prepared in triplicates. The mortality rate of ZF larvae after microinjection was below 10% in all cases.

### 3.5. ZF Sample Preparation and Metabolite Analysis by LC-HRMS/MS

After exposure (see Section 3.3 and Section 3.4), all larvae were transferred into a tube using a pipette and then washed twice with 1 mL of Danieau’s solution. The cleaned larvae were euthanized by placing the tubes in ice water for 15 min. Samples were snap-frozen in liquid nitrogen, followed by lyophilization for 4 h. The lyophilized larvae were stored for a maximum of one week at −20 °C until extraction. For metabolite identification, frozen larvae were thawed at room temperature for at least 30 min and extracted by vigorous vortexing for 2 min with 50 µL methanol. The sample was centrifuged at 10,000× *g* for 2 min at room temperature. All of the supernatant was transferred to an autosampler vial, and 5 µL was injected onto the LC-HRMS/MS system consisting of a Dionex Ultimate 3000 RSLC system (Thermo Fisher Scientific, Germering, Germany) and maXis 4G HR-QTOF mass spectrometer (Bremen, Germany) with the Apollo II ESI source. Separation was carried out on a Waters ACQUITY BEH C_18_ column (100 × 2.1 mm, 1.7 µm) equipped with a Waters VanGuard BEH C_18_ 1.7 µm guard column at 45 °C using 0.1% formic acid in water (*v*/*v*, eluent A) and 0.1% formic acid in acetonitrile (*v*/*v*, eluent B) at a flow rate of 600 µL/min. The linear gradient mode was as follows; 0–0.5 min, 5% eluent B; 0.5–18.5 min, 5–95% eluent B; 18.5–20.5 min, 95% eluent B; 20.5–21 min, 95–5% eluent B; 21–22.5 min, 5% eluent B.

Mass spectra were acquired in centroid mode ranging from 150–2500 *m*/*z* at a 2 Hz full scan rate in the positive ion mode and MS/MS data were collected with automatic precursor selection including the masses of the inclusion list, which was set up for 7′*N*-5F-ADB and its expected metabolite. The precursor ion mass and MS^2^ data (Supplementary Figure S4) were used to confirm the structures of the metabolites [24,27]. Mass spectrometry source parameters were set to 500 V as end plate offset; capillary voltage, 4000 V; nebulizer gas pressure, 1 bar; dry gas flow, 5 L/min, and dry temperature, 200 °C. Ion transfer and quadrupole settings were set to funnel RF 350 Vpp; Multipole RF, 400 Vpp as transfer settings; ion energy, 5 eV as well as a low mass cut of 300 *m*/*z* as quadrupole settings. Collision cell was set to 5 eV; pre-pulse storage time, 5 µs; spectra acquisition rate, 2 Hz. Calibration was carried out automatically before every LC-HRMS/MS run by injection of sodium formate and calibration on the sodium formate clusters forming in the ESI source. All MS analyses were acquired in the presence of the lock masses C_12_H_19_F_12_N_3_O_6_P_3_, C_18_H_19_O_6_N_3_P_3_F_2_, and C_24_H_19_F_36_N_3_O_6_P_3_ which generate the [M + H]^+^ ions of *m*/*z* 622.0290, 922.0098, and 1221.9906. DataAnalysis software version 4.2 (Bruker Daltonics, Bremen, Germany) was used for qualitative analysis. All data were presented as the range of mean and standard deviation (SD) using MS Excel 2016.

Analyzing metabolite data, structural isomers were numbered individually when their corresponding peaks could be separated by chromatography. When isomers where co-eluting, they were analyzed together as one peak as assigned in the tables of this study. In addition, the peak area of each metabolite was taken into consideration when constructing the metabolic pathway of 7′*N*-5F-ADB. Due to the lack of reference standards that would be needed for absolute quantification, our analyses of relative abundances of metabolites relies on the quantification of peak areas, assuming similar ionization behaviors of the individual metabolites.

### 3.6. In Vitro Metabolism Analyses Using HepaRG Cells

To compare the metabolism of an *in vitro* model to ZF larvae, HepaRG cells were investigated as already described by Richter et al. [24]. Undifferentiated cells frozen in a vial were thawed and seeded into T-75 flasks with a dilution factor of 10 (*v*/*v*) at 37 °C in a humidified incubator (95% air humidity, 5% CO_2_). Cell medium was renewed every other day, and cells were sub-cultured to 2 × 10^6^ cells/cm^2^ in T-75 flasks in a total of 15 mL growth medium. After two weeks of proliferation, cells were seeded at a density of 1 × 10^5^ cells/cm^2^ with 2 mL growth medium in 6-well cell culture plates. For the process of cell differentiation, growth medium was replaced with differentiation medium for two more weeks and maintained with medium renewal every second day. Cells for drug metabolism experiments were at passage 18, and all the steps of cell preparation were performed under sterile conditions according to the manufacturer’s instruction (Biopredic International).

Afterwards, the growth medium was composed of MIL 700C (basal hepatic cell medium) supplemented with ADD 720C (differentiation medium supplement with antibiotics), and it was pre-warmed to 37 °C before usage. 500 µL aliquots of medium were removed from the 6-well cell culture plate, and treatment was started by adding 500 µL of 7′*N*-5F-ADB solution. The solutions were prepared by the addition of compound to the growth medium at final concentrations of 20 µM with 0.2% (*v*/*v*) DMSO and 50 µM with 0.5% (*v*/*v*) DMSO.

Treatment was stopped after 0.1, 15, 60, 120, 240, 360, and 1440 min (24 h), and 100 µL-supernatants of medium were transferred into a tube. 100 µL of cold acetonitrile with 0.1% formic acid was immediately added for extraction. Samples were vortexed and cooled in a freezer for 30 min at −20 °C. The samples were centrifuged at 10,000× *g* for 2 min at 4 °C and the supernatant was transferred to an autosampler vial. All samples were then dried *in vacuo* and resuspended with 50 µL of acetonitrile containing 0.1% formic acid. 5 µL was injected onto the LC-HRMS/MS system. All incubation conditions were done in duplicates. Data acquisition and analysis were performed as described in Section 3.5.

### 3.7. MSI Analysis of ZF Larva by MALDI-FT-ICR

Treated ZF larvae (see Section 3.3 and Section 3.4) were directly frozen after embedding in 40% (*w*/*v*) gelatin solution and samples were stored at –20 °C until cryosectioning. Cuts with 10-µm thickness from single larvae were prepared using a cryostat (MEV; SLEE, Mainz, Germany) and they were put on a cold conductive indium-tin-oxide (ITO) coated glass slide. After scanning slides under a microscope to align the optical image of the sample in MALDI, the serial sections from one larva were deposited using TM-Sprayer (HTX M5; HTX Technologies, NC, USA) with 15 mg mL^−1^ 2,5-dihydroxybenzoic acid (2,5-DHB) in acetonitrile:water (9:1, *v*/*v*) containing 0.1% of trifluoroacetic acid, and then dried in a vacuum desiccator for 2 h. The dried glass slide was stored at –20 °C before MSI measurement.

MSI analysis was performed using MALDI and 7.0T SolariX FT-ICR (Bruker Daltonics, Bremen, Germany) in positive ion mode (*m*/*z* range 150–1,000), using 40 laser shots per pixel with a raster width of 20 µm. For auto-calibration of MALDI of each laser measurement, lock mass was set to *m/z* 273.039364 (2,5-DHB matrix), and mass calibration of FT-ICR was carried out using the calibration solution according to the manufacturer’s manual. All MALDI-MSI data acquisitions and image analyses in two dimensions were processed using ftmsControl version. 2.2, flexImaging version 5.0, and SCiLS Lab version 2019b Pro softwares (Bruker Daltonics, Bremen, Germany).

## 4. Summary and Conclusions

ZF larvae were exposed to 7′*N*-5F-ADB by different administration routes and the total number of metabolites observed in these microinjected samples was higher than the number of metabolites detected after conventional waterborne exposure (Table 1). The only exception was the ZF larvae sample, where the NPS was microinjected into yolk sac. Here, only one metabolite was found [24], which we could later explain by the compound’s lack of distribution in metabolically active organs of the larvae. Furthermore, the spatial distribution of 7′*N*-5F-ADB and its metabolites was investigated in detail by MSI and we found significant differences following aquatic exposure and microinjection into different organs (Figure 6 and Figure 7).

Comparing the metabolite pattern from all investigated models (human [27], HepaRG cells, ZF larvae) using LC-HRMS/MS, metabolite M5, formed by ester hydrolysis, was among the most abundant peaks in human samples, HepaRG cells, and all ZF larvae samples prepared in this study. The parent compound was detected in high amounts in all samples except human urine samples, and the metabolites listed as next most abundant were M4 (oxidative defluorination of M5), M30 (glucuronidation of M5), M11 (hydroxylation of M5 in the tertiary butyl part), M13 (oxidation defluorination in combined with oxidation to carboxylic acid), and M16 (hydroxylation of the fluoro pentyl chain isomer 1) (Table 2). These results suggest that the major metabolites were produced sequentially from M5 and the main metabolic reactions were oxidation and hydroxylation as part of phase I metabolism. Interestingly, most of the glucuronidated conjugate metabolites found in human urine were also detected in significant amounts in microinjected ZF larvae (Table 1, Figure 5, Supplementary Figures S1 and S5). Fifteen phase I and phase II metabolites were commonly observed in the three models, which were generated by amide hydrolysis, ester hydrolysis, hydroxylation of the alkyl chains, and glucuronidation (Figure 3a and Table 1).

Remarkably, microinjected ZF larvae had a high concordance rate of 70% to human urine samples, compared to only 55% concordance of HepaRG cell metabolites to human metabolites (Figure 3b). While comparing the mutual similarity of metabolism among the three investigated models, ZF larvae were able to generate four metabolites also found in human urine, which were, however, not detected in HepaRG cells. Encouragingly, 18 out of 20 HepaRG metabolites were also found in ZF larvae, which included 15 compounds that were detected in all three models. It is noteworthy that it could be shown again that ZF larvae produce a metabolite spectrum which is highly similar to human *in vitro* and in vivo metabolism. Furthermore, the metabolic pathway of 7′*N*-5F-ADB [24,27] was refined and extended by addition of the results from ZF larvae and HepaRG cells (Figure 5).

Taken together, among the studied models, microinjected ZF larvae generated a high number of metabolites and the most authentic spectrum of human metabolites of 7′*N*-5F-ADB. Intriguingly, metabolic reactions could be observed already within one hour after microinjection. In contrast, at least 24 h waterborne treatment of ZF larvae was necessary to identify most common metabolites.

Due to its ease of handling and flexibility when it comes to the administration route of compounds, and because of being in accordance with results from human metabolism studies, the ZF larvae model is an excellent in vivo tool for drug metabolism and distribution studies. This is also owing to the fact that we could successfully prepare MS images of treated larvae, which allows us to rationally choose an appropriate route for compound administration. Although further optimization of MSI applied to ZF larvae [33] and the generation of metabolite images need to be done, it is already a unique tool to get a first impression on the distribution of a drug and its metabolites depending on the route of administration. Here, microinjection in vital organs, such as the heart ventricle, appeared to be most beneficial to achieve fast distribution and metabolism of the highly lipophilic 7′*N*-5F-ADB.

## Figures and Tables

**Table 1:** Summary of 7′*N*-5F-ADB and its phase I and II metabolites and their detection in the different models.

Compound		Human Screening Data [27]		Zebrafish Larvae, Published Data [24]		Zebrafish Larvae, Data from This Study			HepaRG In Vitro Model
		Plasma	Urine	Aquatic Exposure	Microinjection				
					Yolk Sac	Caudal Vein	Heart Ventricle	Hindbrain	
Parent compound	7′*N*-5F-ADB	+++	+	+++	++	++	++	++	+++
Phase I	M1		+	+		+	+	+	+
	M2		+	+					+
	M3			+		+	+	+	+
	M4		+	+		+	+	+	+
	M5	++	+++	++		+	+	+	++
	M6		+	+		+	+	+	+
	M7			+		+	+	+	+
	M8		+			+ ^nq^	+ ^nq^	+ ^nq^	
	M9					+ ^nq^	+ ^nq^	+ ^nq^	
	M10		+	+		+	+	+	+
	M11	+	++			+	+ ^nq^	+	+
	M12		+	+		+	+ ^nq^	+	+
	M13		+	+	+++	+++	+++	+++	+
	M14		+						
	M15			+		+	+	+	+
	M16	+		+		+	+	+	+
	M17	+	+			+	+	+	+
	M18		+						
	M19		+						
	M20 ^a^								
	M21		+	+		+	+	+	
	M22								+
	M23			+ ^c^		+ ^b^	+ ^b^	+ ^b^	
	M24		+ ^c^						
	M25		+ ^c^						
	M26								
Total number of Phase I metabolites		4	17	14	1	17	17	17	15
Phase II	M27		+	+		+		+	+
	M28		+			+	+ ^nq^	+	
	M29		+			+	+	+	+
	M30		+	+		+	+	+	+
	M31		+			+	+	+	+
	M32		+						
	M33		+						
	M34		+			+	+	+	
	M35		+ ^c^	+^c^		+ ^b^	+ ^b^	+ ^b^	+ ^b^
	M36		+ ^c^	+^c^					
Total number of Phase II metabolites		-	10	4	-	7	6	7	5
Total number of detected Phase I/II metabolites		4	27	18	1	24	23	24	20

^a^ Precursor metabolite of M26 that was not detected in this study. ^b^ Peaks of structural isomers were not separated in the chromatograms due to co-elution from the LC-HRMS/MS system used in this study, and accordingly, isomers were counted and quantified as one metabolite. ^c^ Isomers of the metabolite eluted as individual peaks using LC-HRMS/MS conditions utilized applied in the previous studies [24,27]. ^nq^ Confirmed mass, but not quantified due to peak detection below signal-to-noise ratio of 3. +: Peak detected, ++: second most abundant peak among metabolites, +++: most abundant peak among metabolites.

**Table 2:** Comparison of the major metabolites detected in the investigated models using LC-HRMS/MS.

	Human Screening Data [27]		Zebrafish Larvae, Published Data [24]			Zebrafish Larvae, Data from this Study		HepaRG In Vitro Model
	Plasma	Urine	Aquatic Exposure ^†^	Microinjection ^††^				
				Yolk Sac	Caudal Vein	Heart Ventricle	Hindbrain	
most abundant peak	P	M5	P	M13	M13	P	M13	P
second most abundant peak	M5	M11	M5	P	P	M5	P	M5
third most abundant peak	M16	M30	M13	-*	M5	M13	M5	M4

P: Parent compound (7′*N*-5F-ADB). * Only one metabolite was produced from zebrafish larvae injected into yolk sac. ^†^ ZF larvae were treated via aquatic exposure from 4 day post-fertilization (dpf) to 5 dpf (1-day exposure) at 28 °C. ^††^ ZF larvae after microinjection of 7′*N*-5F-ADB were incubated at 28 °C for 1 h.

**Table 3:** Comparison of the major metabolites visualized in ZF larva by MALDI-FT-ICR.

	Aquatic Exposure		Microinjection ^†^		
	1-Day Exposure (from 4 dpf to 5 dpf)	3-Day Exposure (from 2 dpf to 5 dpf)	Caudal Vein	Heart Ventricle	Hindbrain
most abundant peak	P	P	M23/M24/ M25/M26	M23/M24/ M25/M26	M23/M24/ M25/M26
second most abundant peak	M13 *	M13	M16/M17	M18/M19/M20	M16/M17
third most abundant peak	M5 *	M5	M18/M19/M20	M16/M17	M18/M19/M20

P: Parent compound (7′*N*-5F-ADB) *. Metabolites were detected at very low abundance and could not be visualized in the sections of ZF larvae. ^†^ Microinjected ZF larvae were incubated at 28 °C for 1 h.

## Supplementary Materials

The following are available online, Figure S1: Detection profile of seventeen minor metabolites of 7′*N*-5F-ADB found in ZF larvae injected into three different organs (caudal vein, heart ventricle, and hindbrain), Figure S2: Internal amount-time profile of five main metabolites (M4, M5, M10, M11, and M30) in HepaRG cells incubated with 20 µM 7′*N*-5F-ADB (*n* = 2), Figure S3: The effects of exposure time and concentration on the formation of five main metabolites [M4, M5, M10, M11, and M30; from (a) to (e)] in HepaRG cells. The main metabolic pathway in HepaRG cells was identified (f), Figure S4: MS^2^ spectra of 11 out of 24 metabolites detected in zebrafish larvae exposed by 7′*N*-5F-ADB, arranged by mass, Figure S5: Schematic representation of 7′*N*-5F-ADB phase I and phase II metabolites in humans, ZF larvae, and HepaRG cells, Table S1: Detailed information of 7′*N*-5F-ADB and its phase I and phase II metabolites in all investigated models.
