# Supplementary material for: Drug Administration Routes Impact the Metabolism of a Synthetic Cannabinoid in the Zebrafish Larvae Model
Source: Molecules. 2020 Sep 29;25(19):4474. doi: 10.3390/molecules25194474 (PMC7582563; doi:10.3390/molecules25194474)
Supplement: Supplementary file 1 [file molecules-25-04474-s001.pdf]

molecules

Supplementary Material

Drug administration routes impact the metabolism of a synthetic cannabinoid in the zebrafish larvae model

Yu Mi Park[1,2], Markus R. Meyer[3], Rolf Müller[1,4,*], and Jennifer Herrmann[1,4,*]

1 Department of Microbial Natural Products, Helmholtz Institute for Pharmaceutical Research Saarland (HIPS), Helmholtz Centre for Infection Research (HZI) and Department of Pharmacy, Saarland University, Campus E8 1, 66123 Saarbrücken, Germany; Yu-Mi.Park@helmholtz-hips.de
2 Environmental Safety Group, Korea Institute of Science and Technology (KIST) Europe, 66123 Saarbrücken, Germany
3 Department of Experimental and Clinical Toxicology, Institute of Experimental and Clinical Pharmacology and Toxicology, Center for Molecular Signaling (PZMS), Saarland University, 66421 Homburg, Germany; m.r.meyer@mx.uni-saarland.de
4 German Center for Infection Research (DZIF), Partner Site Hannover-Braunschweig, Germany
* Correspondence: Rolf.Mueller@helmholtz-hips.de (R.M.); Jennifer.Herrmann@helmholtz-hips.de (J.H.); Tel.: +49-0681-98806-3000 (R.M.), +49-0681-98806-3101 (J.H.)

Received: date; Accepted: date; Published: date

molecules

MDPI

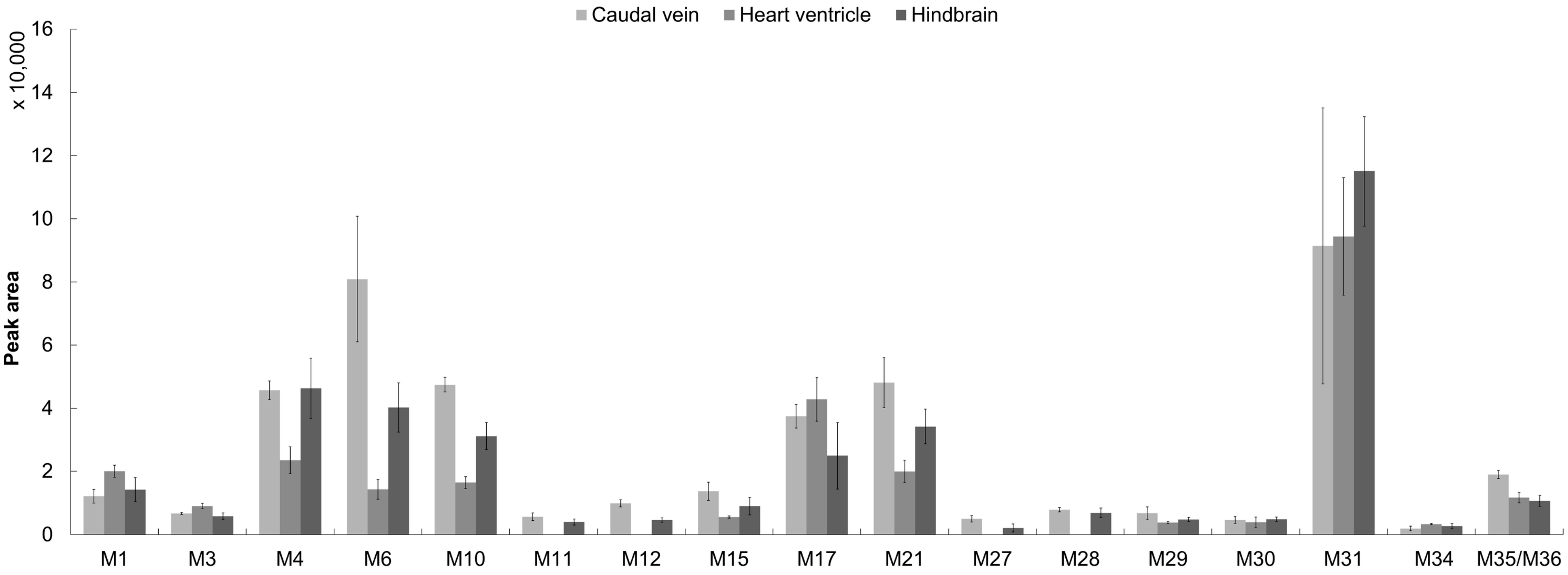

Figure S1. Detection profile of seventeen minor metabolites of 7'*N*-5F-ADB found in ZF larvae injected into three different organs (caudal vein, heart ventricle, and hindbrain). M11, M12, and M28 in the heart ventricle samples were observed with a low peak detection below signal-to-noise (S/N) ratio of 3, which resulted in no detectable peak area in this graph, but there was no detection of M27. Both, M8 and M9 in all microinjected ZF larvae were not quantified due to the low detection below S/N ratio of 3. The clustered columns are displayed as mean ± SD (n=3).

molecules

MDPI

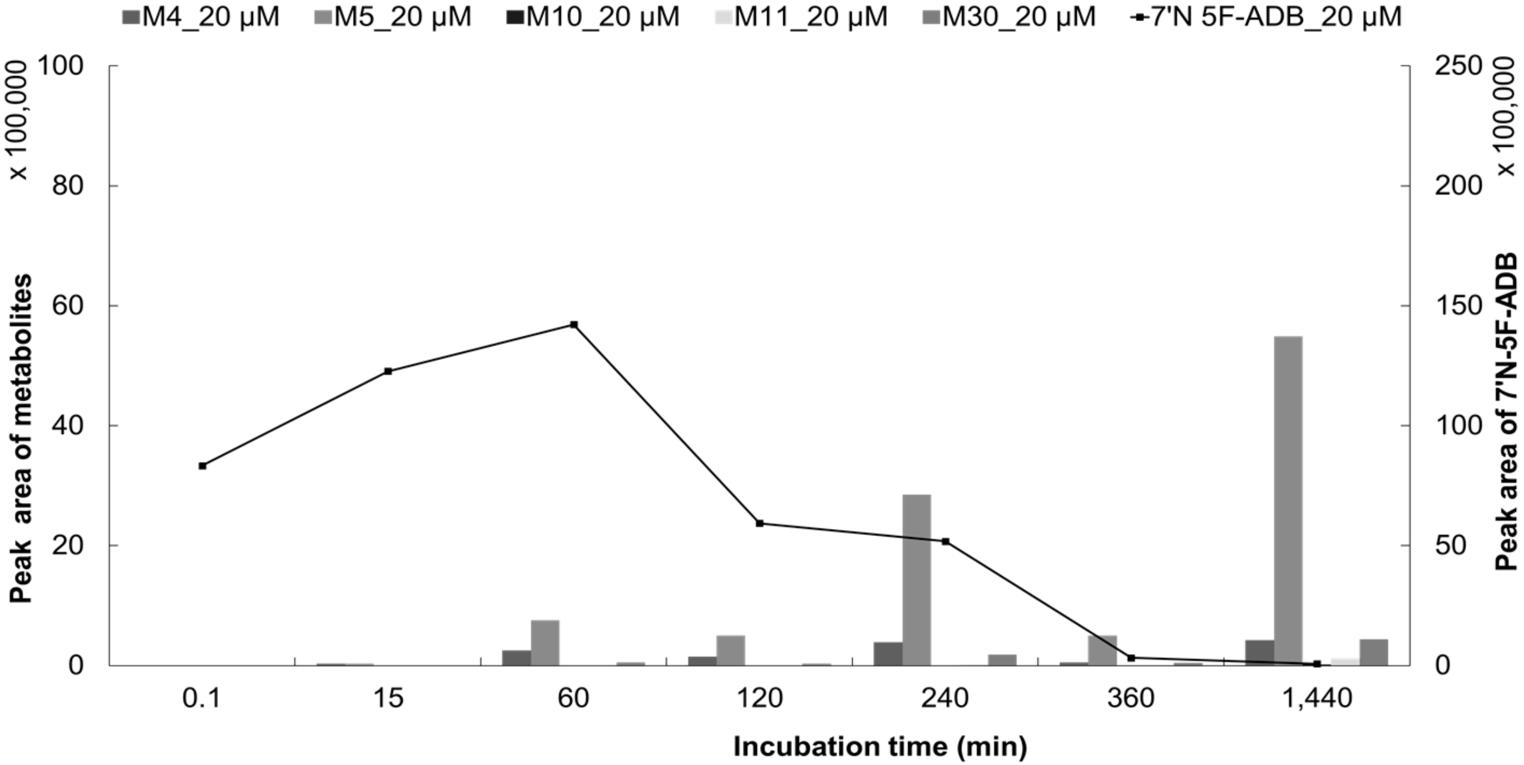

(a)

Figure S2. *Cont.*

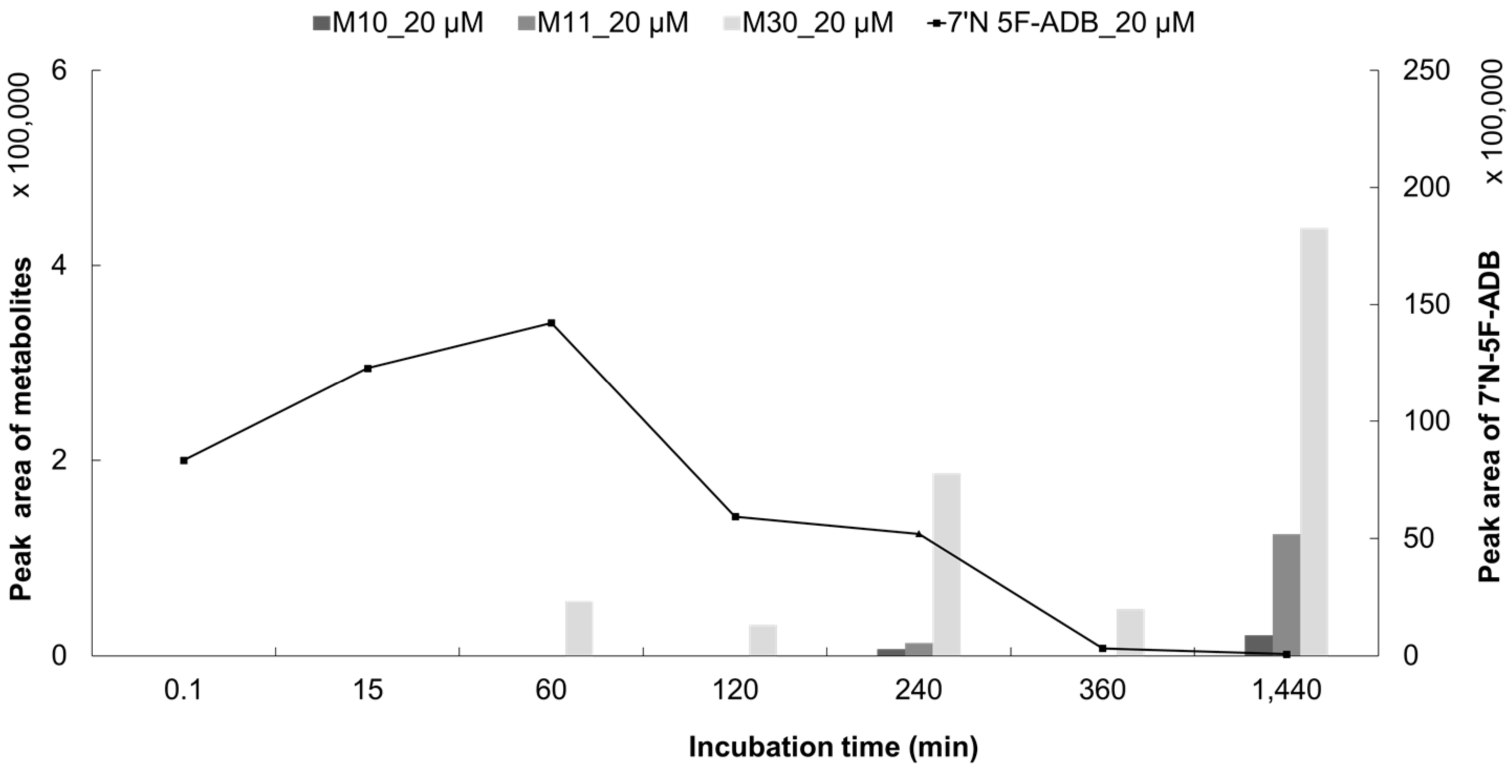

(b)

Figure S2. ***Cont.***

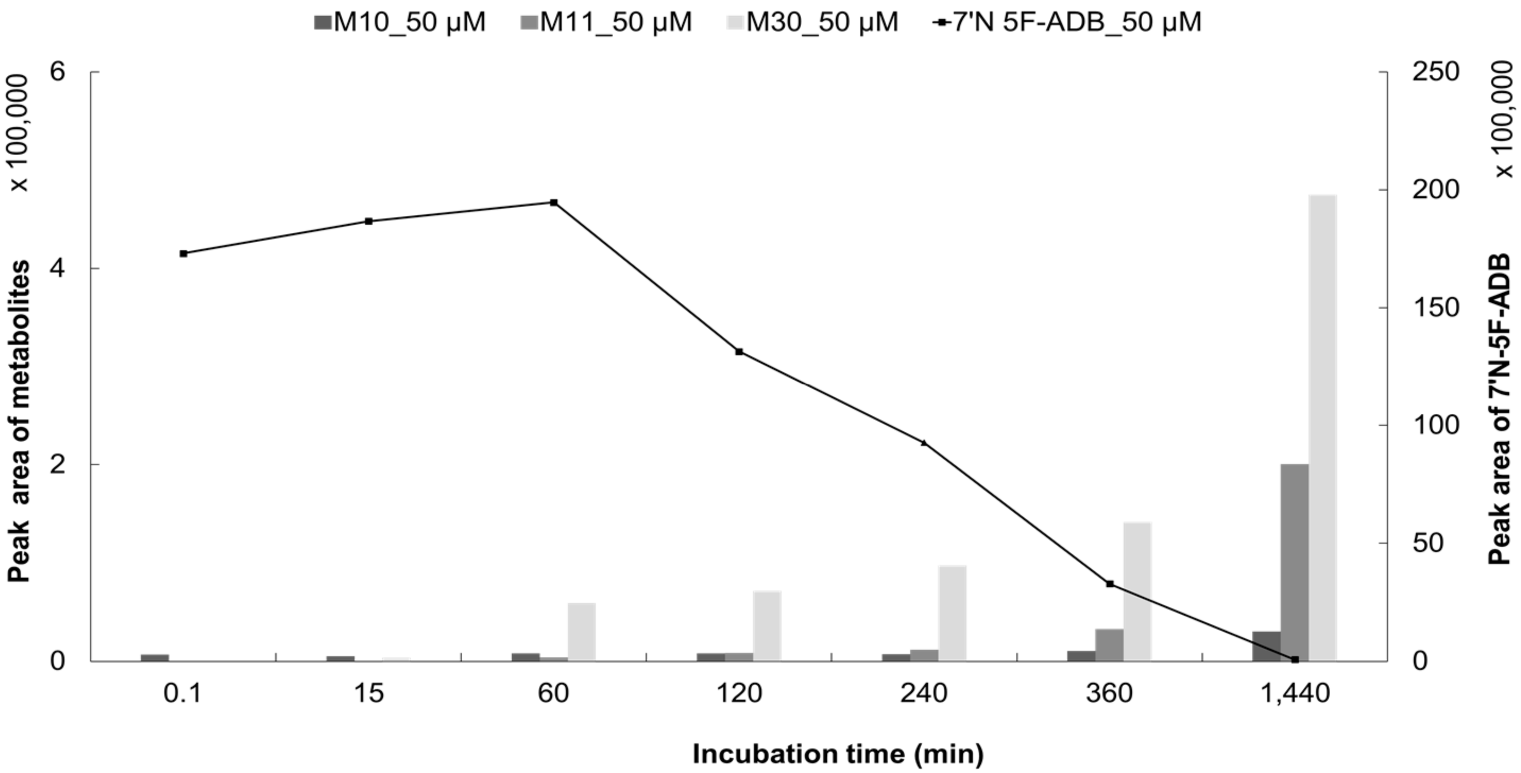

(c)

Figure S2. Internal amount-time profile of five main metabolites (M4, M5, M10, M11, and M30) in HepaRG cells incubated with 20 µM 7'*N*-5F-ADB (a) (n=2). (b) and (c) are the internal amount-time profiles of metabolites M10, M11, and M30. Incubation with 20 µM 7'*N*-5F-ADB (b) resulted in overall lower peak areas than incubation with 50 µM 7'*N*-5F-ADB (c).

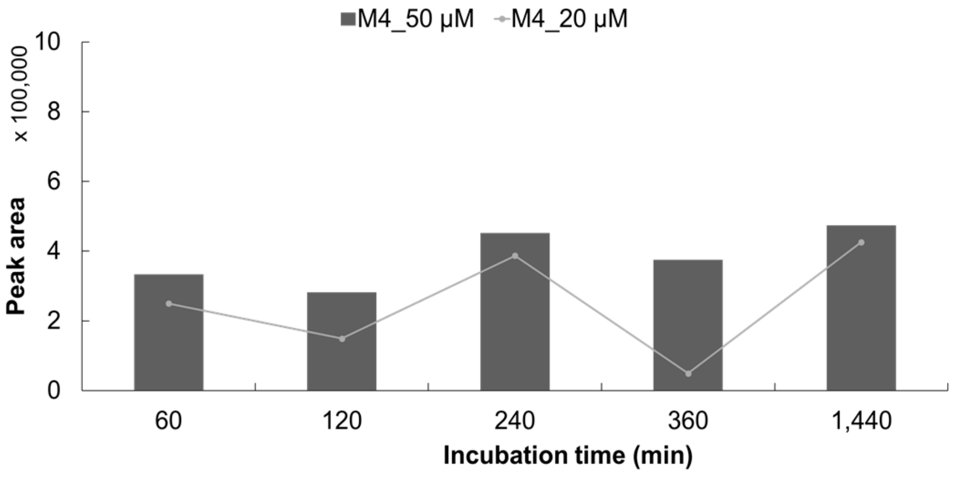

(a)

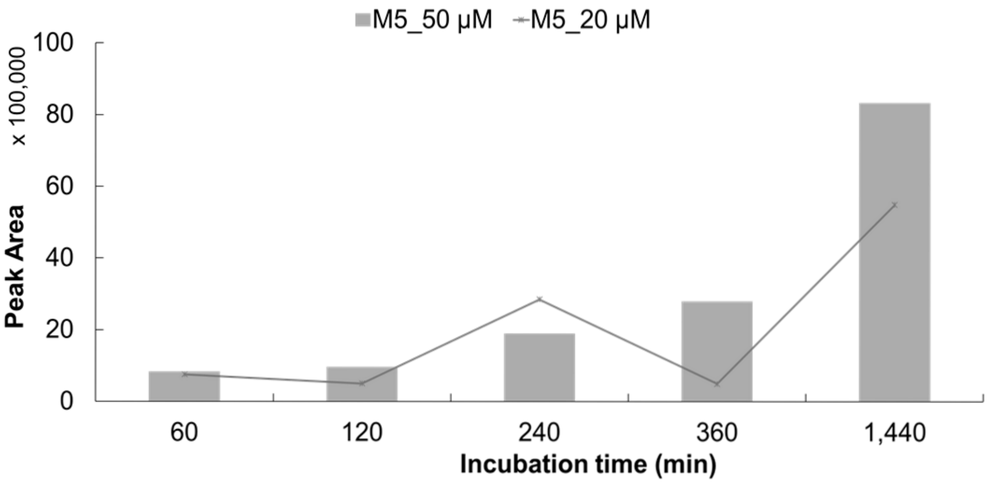

(b)

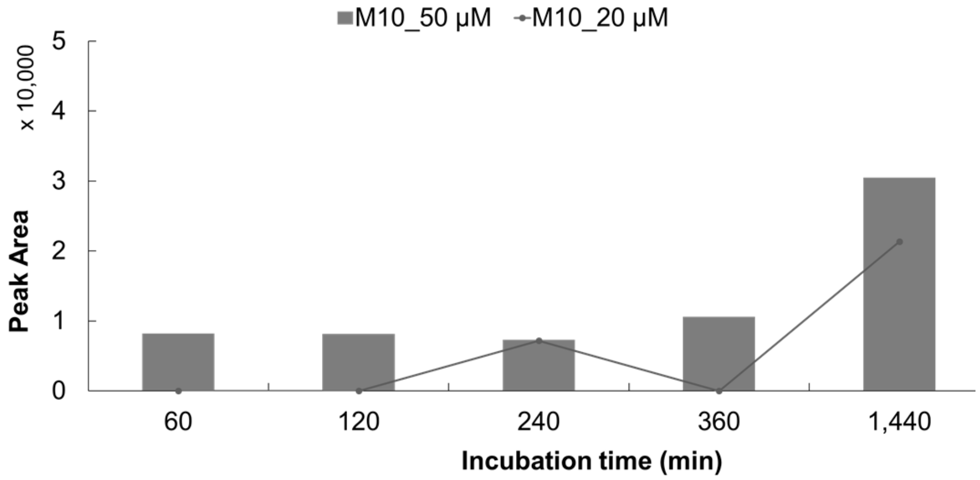

(c)

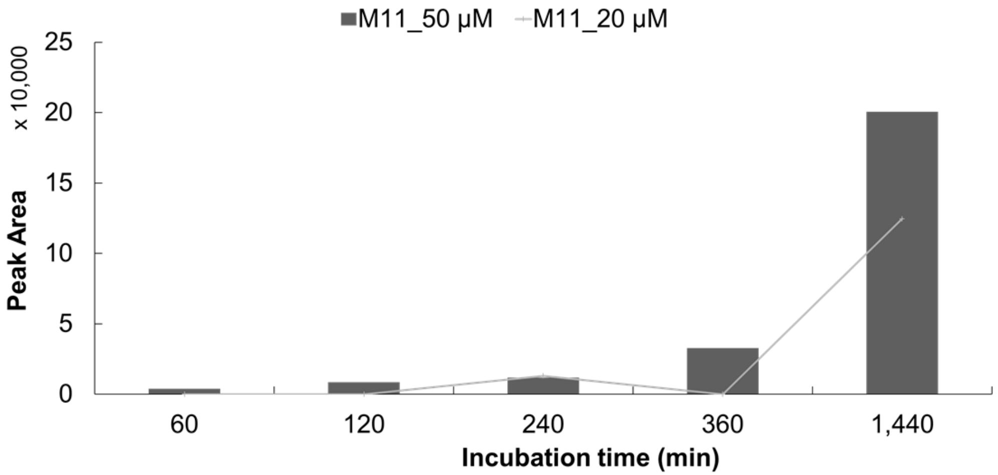

(d)

Figure S3. ***Cont.***

molecules

MDPI

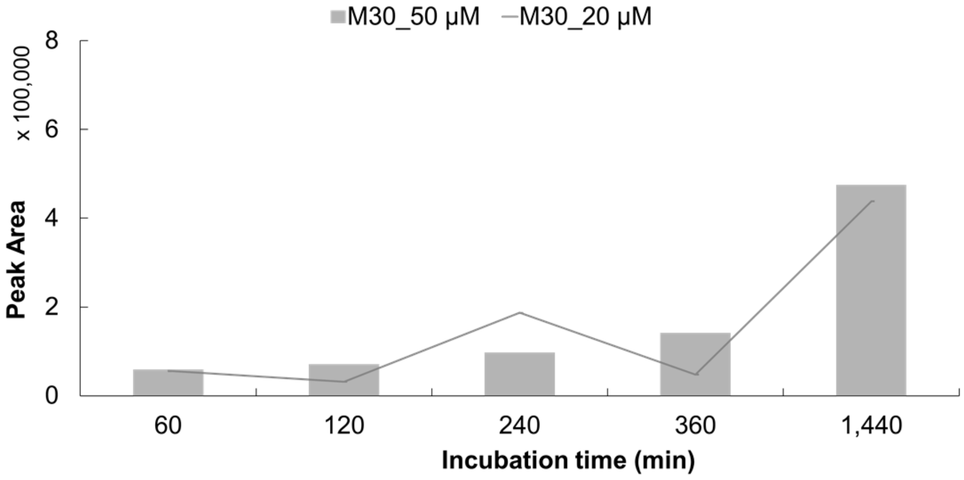

(e)

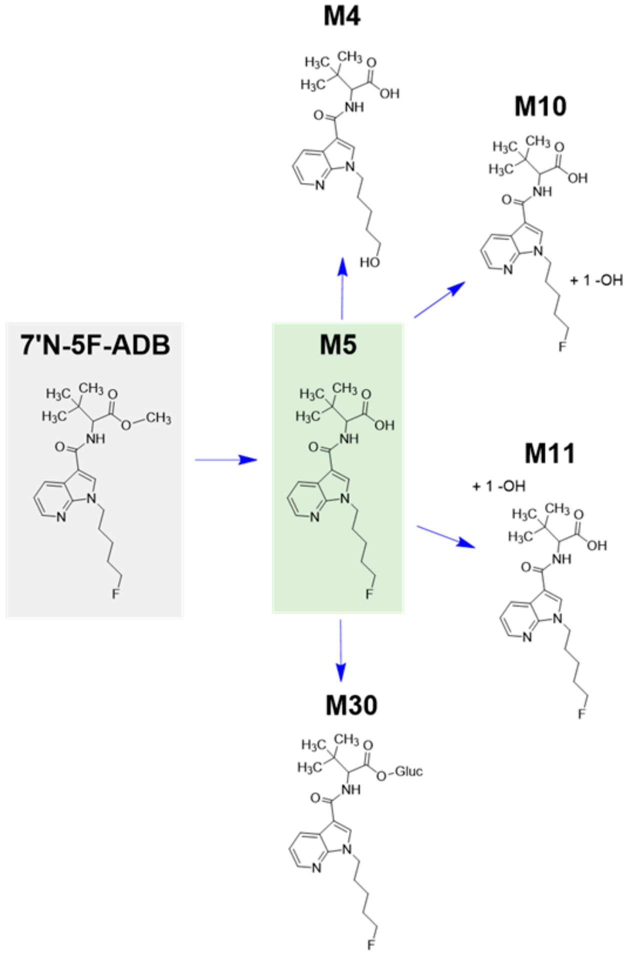

(f)

Figure S3. The effects of exposure time and concentration on the formation of five main metabolites (M4, M5, M10, M11, and M30; from (a) to (e)) in HepaRG cells. These graphs show the peak area of each metabolite during the incubation time from 60 min to 1,440 min following treatment with 20 μM (marked lines) and 50 μM (clustered columns) 7′*N*-5F-ADB. The main metabolic pathway in HepaRG cells was identified (f).

molecules
MDPI

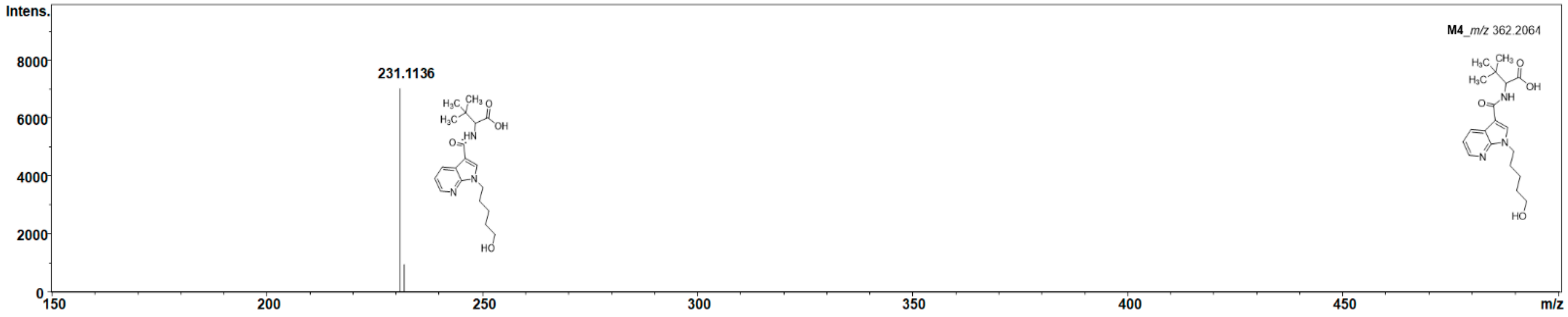

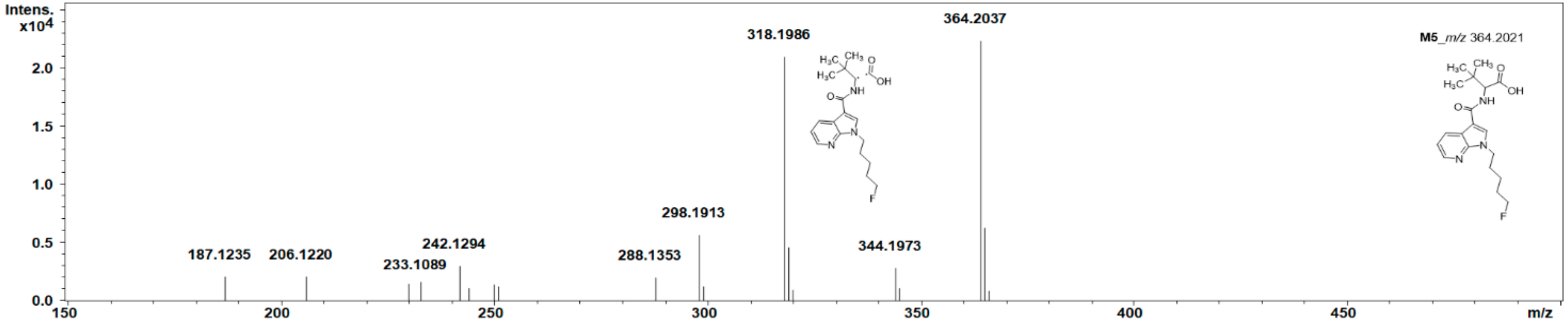

Figure S4. *Cont.*

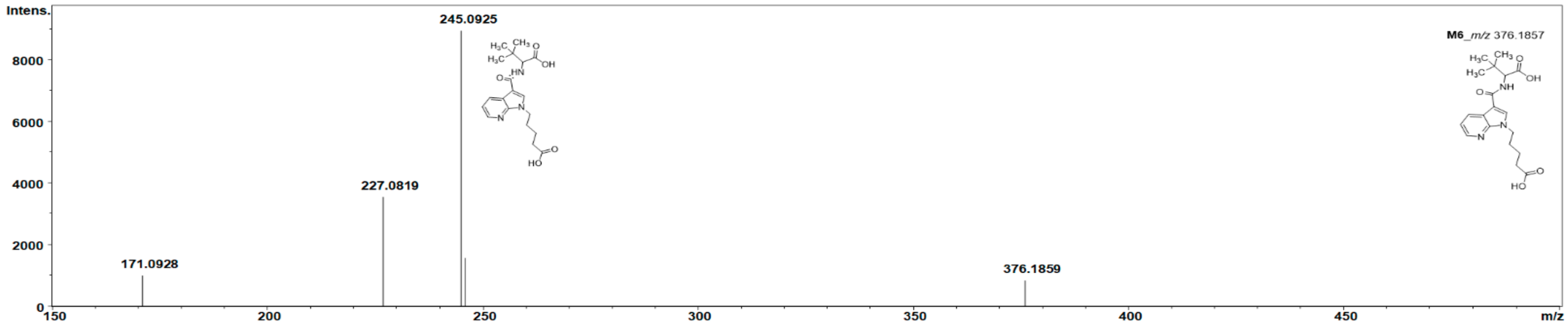

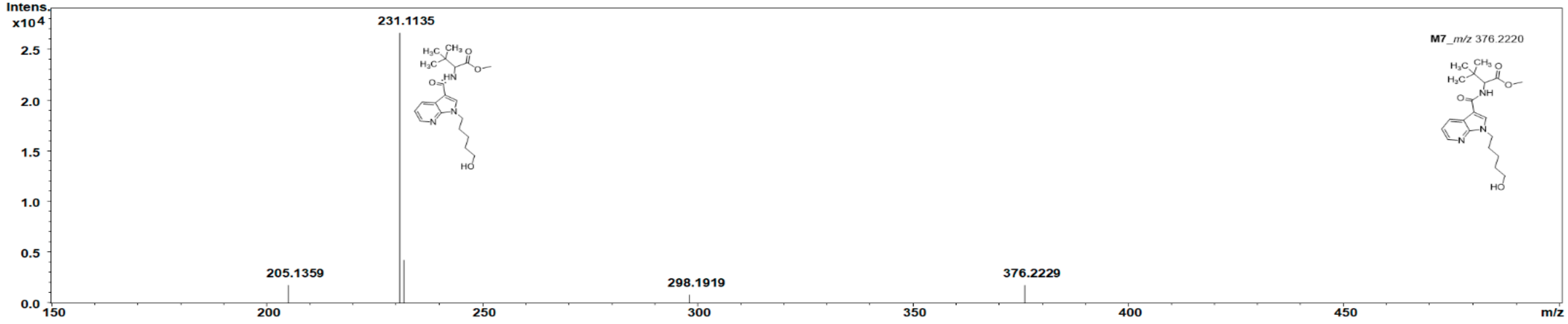

Figure S4. ***Cont.***

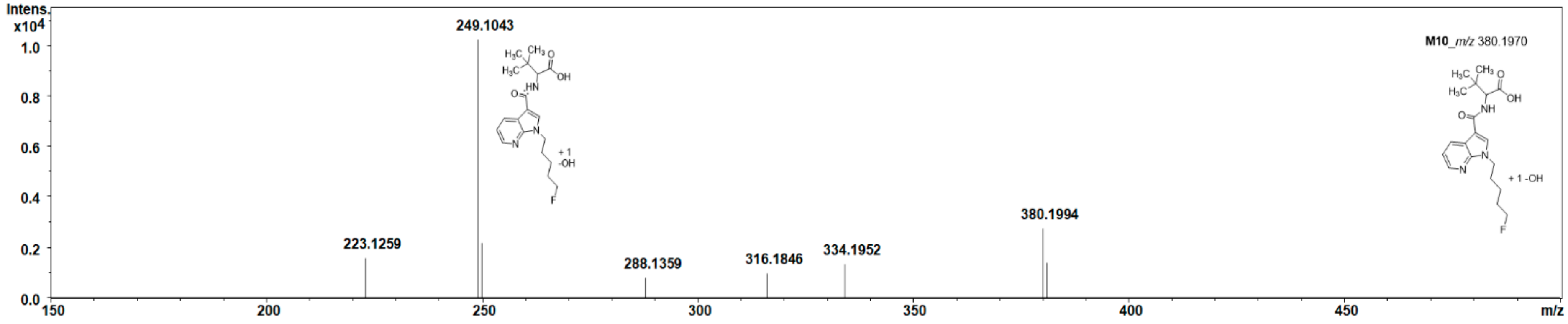

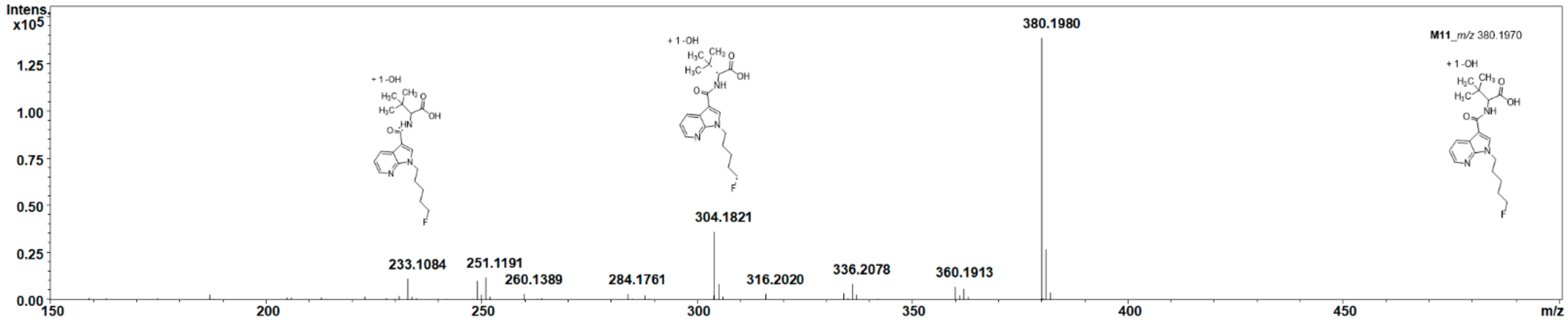

Figure S4. ***Cont.***

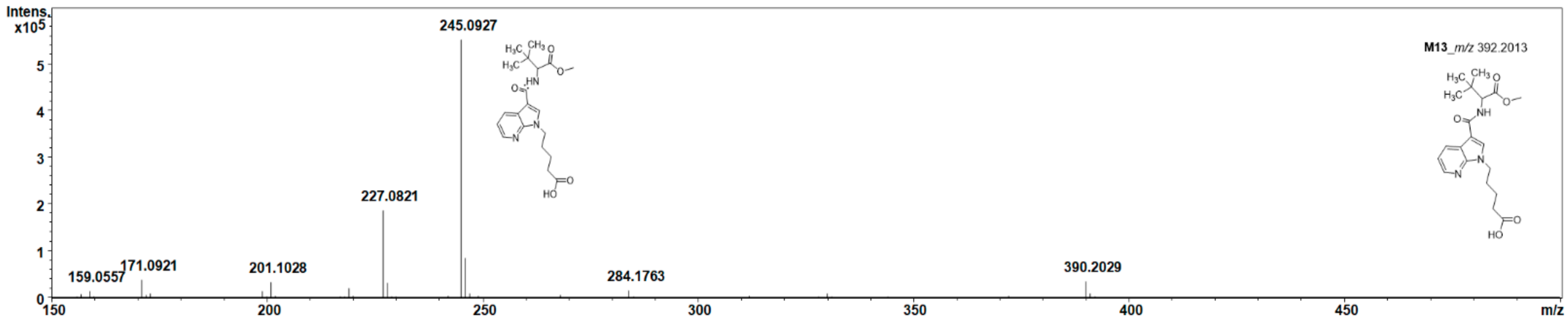

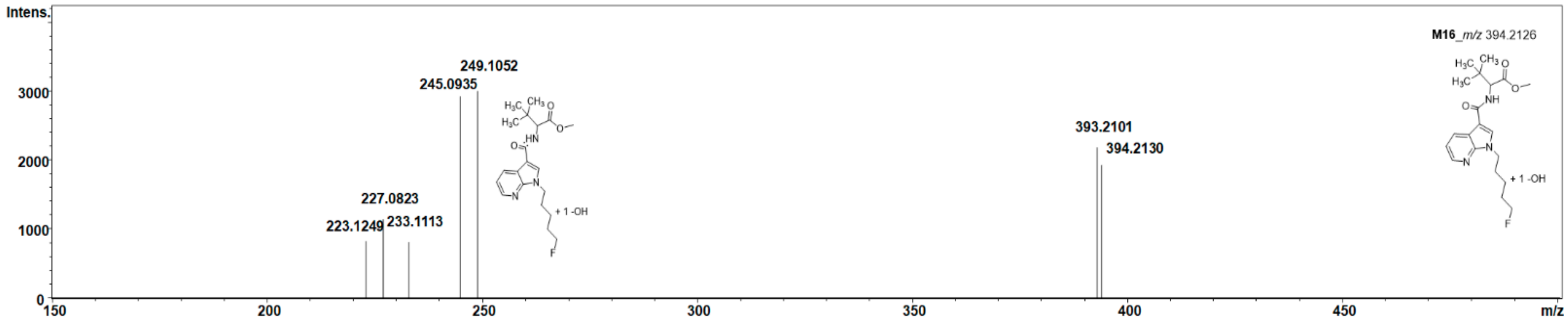

Figure S4. ***Cont.***

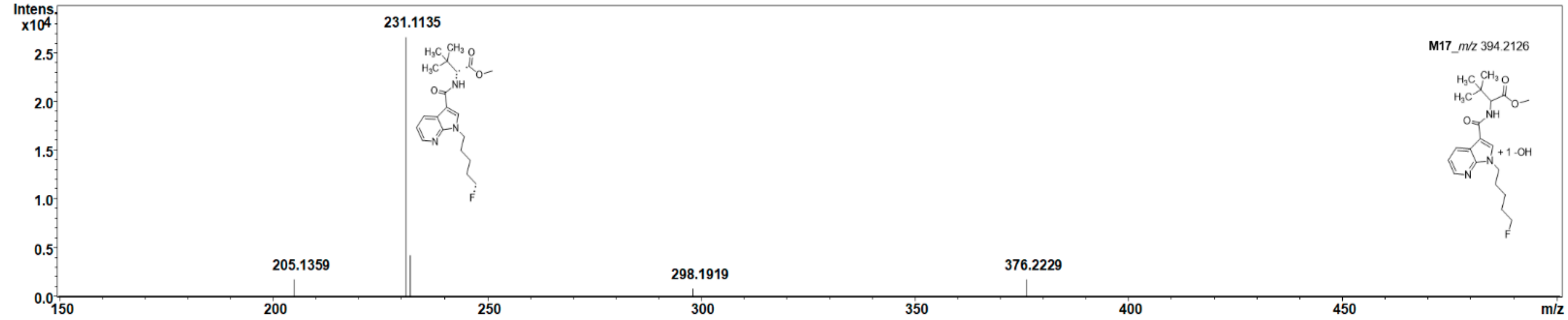

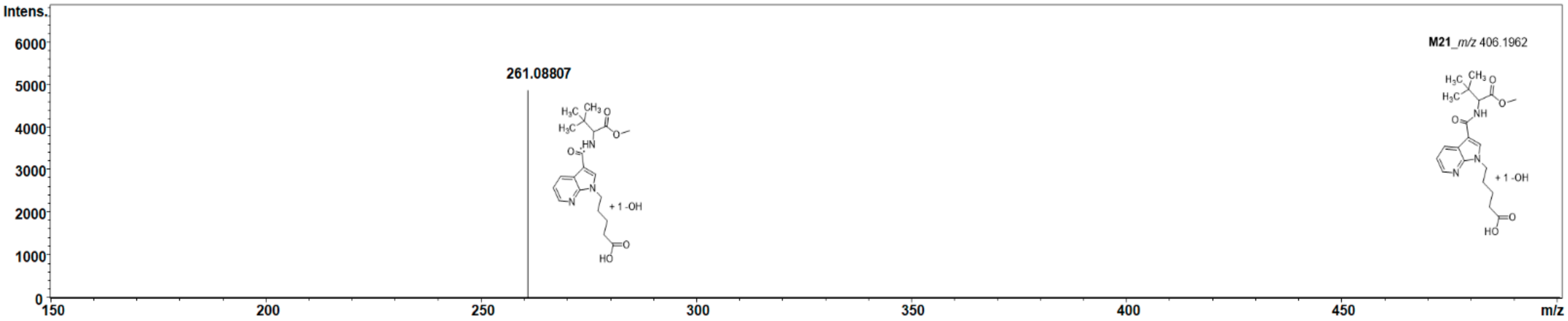

Figure S4. ***Cont.***

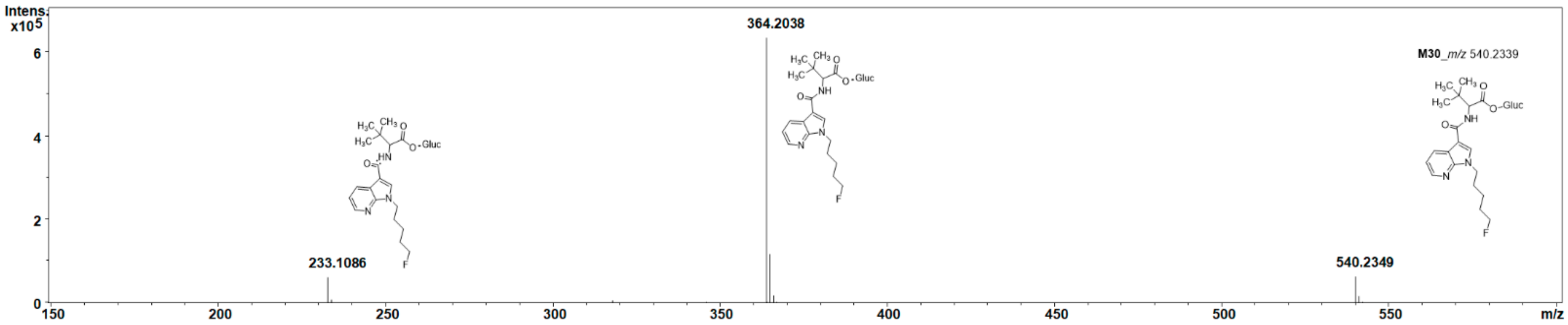

Figure S4. MS^2 spectra of 11 out of 24 metabolites detected in zebrafish larvae exposed to 7′*N*-5F-ADB, arranged by mass. The tentative structures of other metabolites, which are not displayed here, were confirmed by MS/MS^2 data in a published study [24, 27].

molecules MDPI

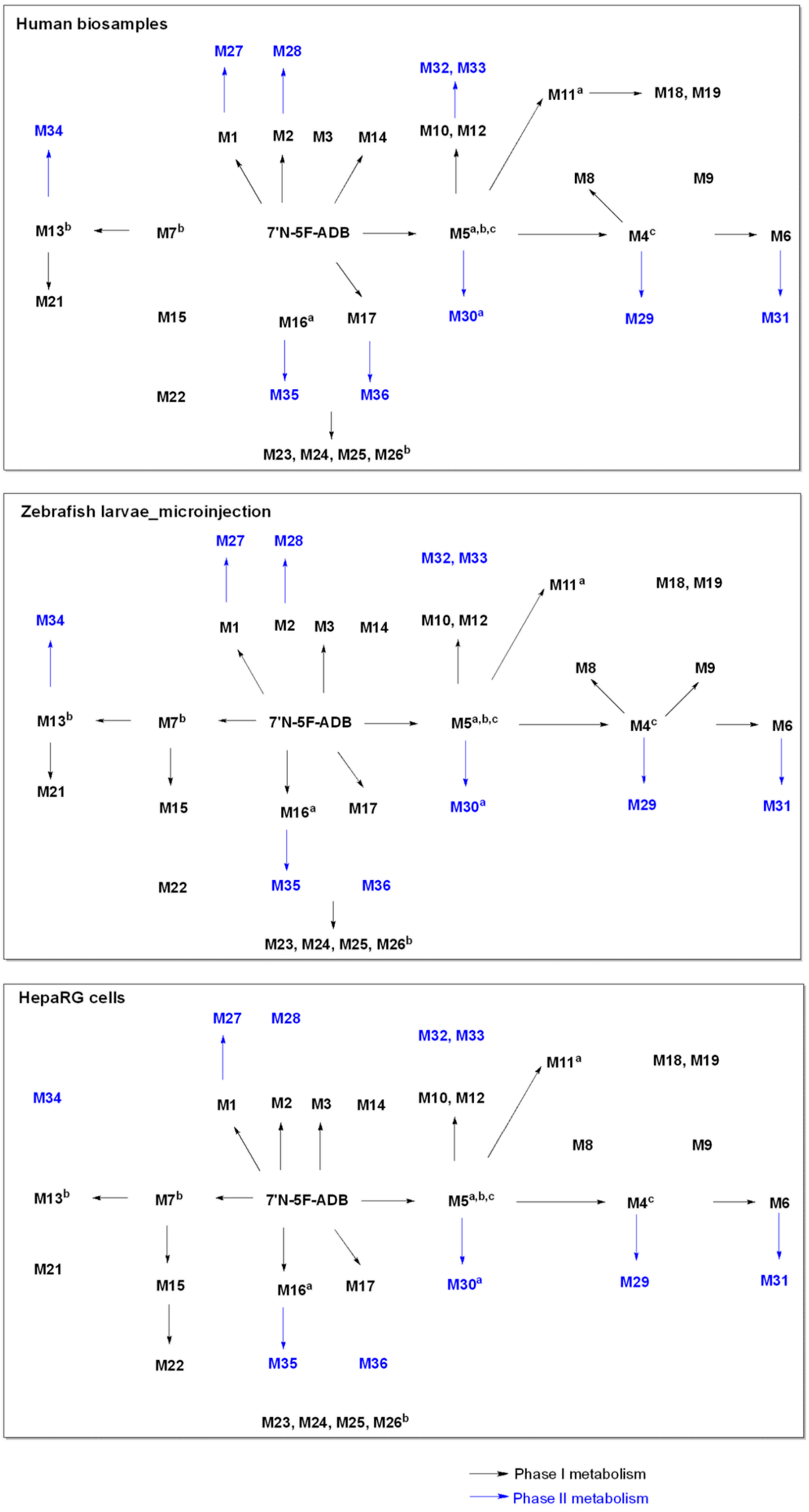

Figure S5. Schematic representation of 7′*N*-5F-ADB phase I and phase II metabolites in humans, ZF larvae, and HepaRG cells. [a]Major metabolites in human samples; [b]major metabolites in microinjected ZF larvae; [c]major metabolites in HepaRG cells.

molecules

MDPI

Table S1. Detailed information of 7′*N*-5F-ADB and its phase I and phase II metabolites in all investigated models.

Compound		Calculated exact masses (*m*/*z*)	Metabolic reaction	Human Screening Data [27]		Zebrafish Larvae, Published Data [24]	Zebrafish Larvae, Data from this Study				HepaRG *In Vitro* Model
							Microinjection				
				Plasma	Urine	Aquatic Exposure	Yolk sac	Caudal Vein	Heart Ventricle	Hindbrain	
Parent compound	7′*N*-5F-ADB	378.2177	Parent compound	+++	+	+++	++	++	++	++	+++
Phase I	M1	251.1184	Amide hydrolysis		+	+		+	+	+	+
	M2	276.1335	Ester hydrolysis + *N*-dealkylation		+	+					+
	M3	290.1941	*N*-dealkylation			+		+	+	+	+
	M4	362.2064	Ester hydrolysis + oxidative defluorination		+	+		+	+	+	+
	M5	364.2021	Ester hydrolysis	++	+++	++		+	+	+	++
	M6	376.1857	Ester hydrolysis + oxidative defluorination + oxidation to carboxylic acid		+	+		+	+	+	+
	M7	376.2220	Oxidative defluorination			+		+	+	+	+
	M8	378.2013	Ester hydrolysis + oxidative defluorination + hydroxylation of the tertiary butyl part isomer 1		+			+^{nq}	+^{nq}	+^{nq}	
	M9	378.2013	Ester hydrolysis + oxidative defluorination + hydroxylation of the pentyl chain isomer 2					+^{nq}	+^{nq}	+^{nq}	
	M10	380.1970	Ester hydrolysis + hydroxylation of the fluoro pentyl chain isomer 1		+	+		+	+	+	+
	M11	380.1970	Ester hydrolysis + hydroxylation of the tertiary butyl part isomer 2	+	++			+	+^{nq}	+	+
	M12	380.1970	Ester hydrolysis + hydroxylation of the fluoro pentyl chain isomer 3		+	+		+	+^{nq}	+	+

	M13	390.2013	Oxidative defluorination + oxidation to carboxylic acid		+	+	+++	+++	+++	+++	+
	M14	392.1806	Ester hydrolysis + oxidative defluorination + oxidation to carboxylic acid + hydroxylation of the tertiary butyl part		+						
	M15	392.2169	Oxidative defluorination + hydroxylation of the pentyl chain			+		+	+	+	+
	M16	394.2126	Hydroxylation of the fluoro pentyl chain isomer 1	+		+		+	+	+	+
	M17	394.2126	Hydroxylation of the pyrrolo pyridine part isomer 2	+	+			+	+	+	+
	M18	396.1919	Ester hydrolysis + dihydroxylation of the fluoro pentyl chain and tertiary butyl part isomer 1		+						
	M19	396.1919	Ester hydrolysis + dihydroxylation of the fluoro pentyl chain and tertiary butyl part isomer 2		+						
	M20 [a]	396.1919	Ester hydrolysis + dihydroxylation of the fluoro pentyl chain isomer 3								
	M21	406.1962	Oxidative defluorination + oxidation to carboxylic acid + hydroxylation of the pentyl chain		+	+		+	+	+	
	M22	408.2118	Oxidative defluorination + dihydroxylation of the pentyl chain								+
	M23	410.2075	Dihydroxylation of the fluoro pentyl chain and tertiary butyl part isomer 1			+ [c]					
	M24	410.2075	Dihydroxylation of the pyrrolo pyridine part and tertiary butyl part isomer 2		+ [v]			+ [b]	+ [b]	+ [b]	
	M25	410.2075	Dihydroxylation of the fluoro pentyl chain and pyrrolo pyridine part isomer 3		+ [c]						
	M26	410.2075	Dihydroxylation of the fluoro pentyl part isomer 4								
**Total number of phase I metabolites**				4	17	14	1	17	17	17	15
Phase II	M27	427.1502	Amid hydrolysis + glucuronidation		+	+		+		+	+
	M28	452.1653	Ester hydrolysis + *N*-dealkylation + glucuronidation		+			+	+ [nq]	+	
	M29	538.2382	Ester hydrolysis + oxidative defluorination + glucuronidation		+			+	+	+	+
	M30	540.2339	Ester hydrolysis + glucuronidation		+	+		+	+	+	+

M31	552.2175	Ester hydrolysis + oxidative defluorination + oxidation to carboxylic acid + glucuronidation		+			+	+	+	+
M32	556.2288	Ester hydrolysis + hydroxylation of the fluoro pentyl chain + glucuronidation isomer 1		+						
M33	556.2288	Ester hydrolysis + hydroxylation of the fluoro pentyl chain + glucuronidation isomer 2		+						
M34	566.2331	Oxidative defluorination + oxidation to carboxylic acid + glucuronidation		+			+	+	+	
M35	570.2444	Hydroxylation of the fluoro pentyl chain + glucuronidation isomer 1		+ [c]	+ [c]		+ [b]	+ [b]	+ [b]	+ [b]
M36	570.2444	Hydroxylation of the pyrrolo pyridine part + glucuronidation isomer 2		+ [c]	+ [c]					
**Total number of phase II metabolites**			-	10	4	-	7	6	7	5
**Total number of detected phase I/II metabolites**			**4**	**27**	**18**	**1**	**24**	**23**	**24**	**20**

[a] Precursor metabolite of M20 that was not detected in this study. [b] Peaks of structural isomers were not separated in the chromatograms due to co-elution from the LC-HRMS/MS system used in this study, and accordingly, isomers were counted and quantified as one metabolite. [c] Isomers of the metabolite eluted as individual peaks using LC-HRMS/MS conditions utilized applied in the previous studies [24, 27]. [nq] Confirmed mass, but not quantified due to peak detection below signal-to-noise ratio of 3. +: Peak detected, ++: second most abundant peak among metabolites, +++: most abundant peak among metabolites.
